# PROTAC-mediated NR4A1 degradation as a novel strategy for cancer immunotherapy

**DOI:** 10.1084/jem.20231519

**Published:** 2024-02-09

**Authors:** Lei Wang, Yufeng Xiao, Yuewan Luo, Rohan P. Master, Jiao Mo, Myung-Chul Kim, Yi Liu, Chandra K. Maharjan, Urvi M. Patel, Umasankar De, Madison E. Carelock, Tanzia Islam Tithi, Xiangming Li, Donald R. Shaffer, Kevin R. Guertin, Haoyang Zhuang, Emily Moser, Keiran S.M. Smalley, Dongwen Lv, Daohong Zhou, Guangrong Zheng, Weizhou Zhang

**Affiliations:** 1 Department of Pathology, Immunology and Laboratory Medicine, College of Medicine, University of Florida, Gainesville, FL, USA; 2 Department of Medicinal Chemistry, College of Pharmacy, University of Florida, Gainesville, FL, USA; 3 Veterinary Diagnostic Laboratory Medicine, College of Veterinary Medicine, Jeju National University, Jeju-si, South Korea; 4 Sanofi Oncology, Sanofi, Cambridge, MA, USA; 5 Sanofi Integrated Drug Discovery, Sanofi, Cambridge, MA, USA; 6 Rheumatology and Clinical Immunology, Department of Medicine, College of Medicine, University of Florida, Gainesville, FL, USA; 7 Division of Pulmonary, Critical Care and Sleep Medicine, Department of Medicine, College of Medicine, University of Florida, Gainesville, FL, USA; 8 Department of Tumor Biology, Moffitt Cancer Center and Research Institute, Tampa, FL, USA; 9 Department of Biochemistry and Structural Biology, Center of Innovative Drug Discovery, University of Texas Health Science Center at San Antonio, San Antonio, TX, USA; 10 University of Florida Health Cancer Center, University of Florida, Gainesville, FL, USA

## Abstract

An effective cancer therapy requires killing cancer cells and targeting the tumor microenvironment (TME). Searching for molecules critical for multiple cell types in the TME, we identified NR4A1 as one such molecule that can maintain the immune suppressive TME. Here, we establish NR4A1 as a valid target for cancer immunotherapy and describe a first-of-its-kind proteolysis-targeting chimera (PROTAC, named NR-V04) against NR4A1. NR-V04 degrades NR4A1 within hours in vitro and exhibits long-lasting NR4A1 degradation in tumors with an excellent safety profile. NR-V04 inhibits and frequently eradicates established tumors. At the mechanistic level, NR-V04 induces the tumor-infiltrating (TI) B cells and effector memory CD8^+^ T (Tem) cells and reduces monocytic myeloid-derived suppressor cells (m-MDSC), all of which are known to be clinically relevant immune cell populations in human melanomas. Overall, NR-V04–mediated NR4A1 degradation holds promise for enhancing anticancer immune responses and offers a new avenue for treating various types of cancers such as melanoma.

## Teaser

The study introduces a new immunotherapy for treating melanoma and other cancers by developing a PROTAC that degrades NR4A1, an intracellular nuclear factor that plays a crucial role in immune suppression.

## Graphical Abstract

**Figure:**
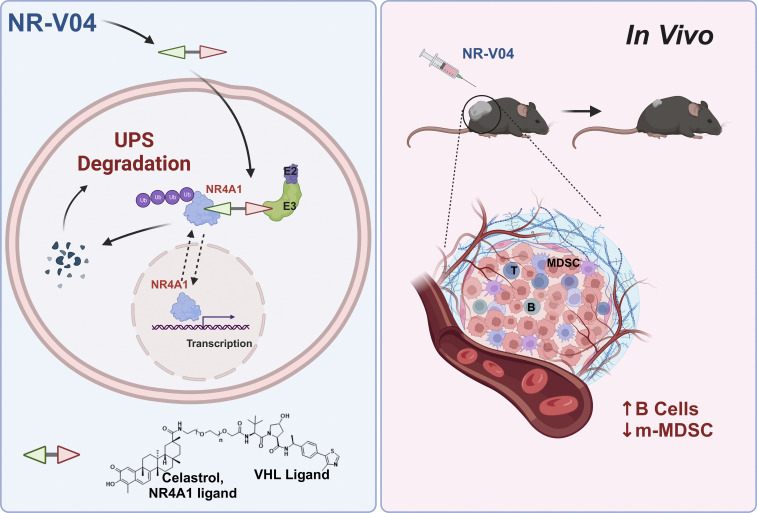

## Introduction

The tumor microenvironment (TME) consists of many cell types that cooperatively promote tumor development and progression. Most cancer therapeutics are designed to target one molecule in one defined cell type. For example, vemurafenib (BRAF inhibitor) inhibits melanoma through targeting mutated BRAF, whereas pembrolizumab (anti–PD-1 antibody) blocks the immune checkpoint PD-1 on T cells to increase antitumor immunity. Several FDA-approved combination therapies involve cancer cell–killing chemotherapies and immune checkpoint inhibitors (ICI) to activate anticancer immunity within the TME, suggesting that cotargeting cancer cells and other cell types within the TME can be effective therapeutic regimens.

NR4A1 is an intracellular transcription factor that is known for its role in immune regulation and other functions (Fassett et al., 2012; Hiwa et al., 2021; Liu et al., 2019; Tan et al., 2020). Within the TME, NR4A1 acts upon several cell types, as we recently summarized (Carelock et al., 2023): (1) NR4A1 is involved in angiogenesis in the B16 melanoma model (Zeng et al., 2006). The impact of NR4A1 on neoangiogenesis has also been confirmed in several other models (Chen et al., 2020; Ha et al., 2008; Ismail et al., 2012; Ye et al., 2019). As neoangiogenesis in tumors induces the formation of new blood vessels with increased permeability, NR4A1 plays a critical role in regulating basal vascular permeability by increasing endothelial nitric-oxide synthase and downregulating several junction proteins involved in adherens junctions and tight junctions (Zhao et al., 2011). (2) The NR4A family has been shown to be elevated in exhausted CD8^+^ T cells (Texh), and their deletion rescues the cytotoxicity function of CD8^+^ T cells (Chen et al., 2019a; Liu et al., 2019). (3) Tumor-infiltrating (TI) regulatory T (Treg) cells rely on NR4A family members for their immune suppressive function (Fassett et al., 2012; Hibino et al., 2018). (4) NR4A1 can be induced in TI natural killer (TI-NK) cells through the IFN-γ/STAT1/IRF1 signaling pathway, which leads to diminished NK cell–mediated cytotoxicity against hepatocellular carcinoma (Yu et al., 2022). (5) The role of NR4A1 in TI-B cells is unknown. Under a different context, NR4A1 limits the expansion of B cells in response to antigen stimulation when secondary signals are lacking and constrains the survival of self-reactive B cells in peripheral tissues (Tan et al., 2020). Some studies have indicated that regulatory B (Breg) cells correlate with shorter survival times in patients, such as bladder cancer (Welinder et al., 2016). There are more studies suggesting that a higher intratumoral density of B cells is associated with good prognosis and improved sensitivity to ICIs in primary cutaneous melanomas (Garg et al., 2016) and metastatic melanomas (Cabrita et al., 2020; Griss et al., 2019). The broader involvement of NR4A1 across different immune cells, such as T, B, and NK cells, underlines its multifaceted roles in the tumor-associated immune response.

Several NR4A1 antagonists have been discovered, such as celastrol, a triterpenoid quinine methide derived from the root of *Tripterygium wilfordii* (Corson and Crews, 2007), which covalently binds to NR4A1 and possesses anti-inflammatory properties (Chen et al., 2019b; Hu et al., 2017; Zhang et al., 2018). Celastrol is not an ideal candidate for clinical development due to limitations such as low bioavailability, a narrow therapeutic window, and undesired adverse effects (Shi et al., 2020). Nevertheless, the moderate binding affinity of celastrol to NR4A1 (Kd of 0.29 μM) (Hu et al., 2017) makes it a promising starting point as a warhead for the design and synthesis of proteolysis-targeting chimeras (PROTACs) targeting NR4A1. PROTACs are bifunctional molecules that consist of a pharmacophore targeting a protein of interest and another pharmacophore recruiting an E3 ligase such as von Hippel Lindau (VHL) or cereblon, facilitating polyubiquitination and subsequent proteasome degradation of the target protein (Békés et al., 2022; Schapira et al., 2019). Unlike the conventional “occupancy-driven” inhibitor-based therapy, PROTACs work in a catalytic and “event-driven” manner, leading to the degradation of protein targets. This unique mechanism of action offers several advantages including higher potency, prolonged pharmacological effects, and improved selectivity. More importantly, PROTACs offer new possibilities to target traditionally undruggable or difficult-to-drug targets, such as transcriptional factors, scaffolding proteins, and multifunctional proteins that cannot be effectively inhibited by a single inhibitor.

Here, we report a first-of-its-kind NR4A1-targeting PROTAC named NR-V04 as a lead for cancer therapy. We performed extensive characterization of NR-V04 and revealed it to have a promising pharmacokinetic (PK) and pharmacodynamic (PD) profile, degradation efficiency and specificity for NR4A1, tumor-inhibitory effect, and immune activation within the TME, along with its low toxicity within the therapeutic dosing range.

## Results

### NR4A1 is a valid target in the TME for cancer therapy

**Figure 1.**
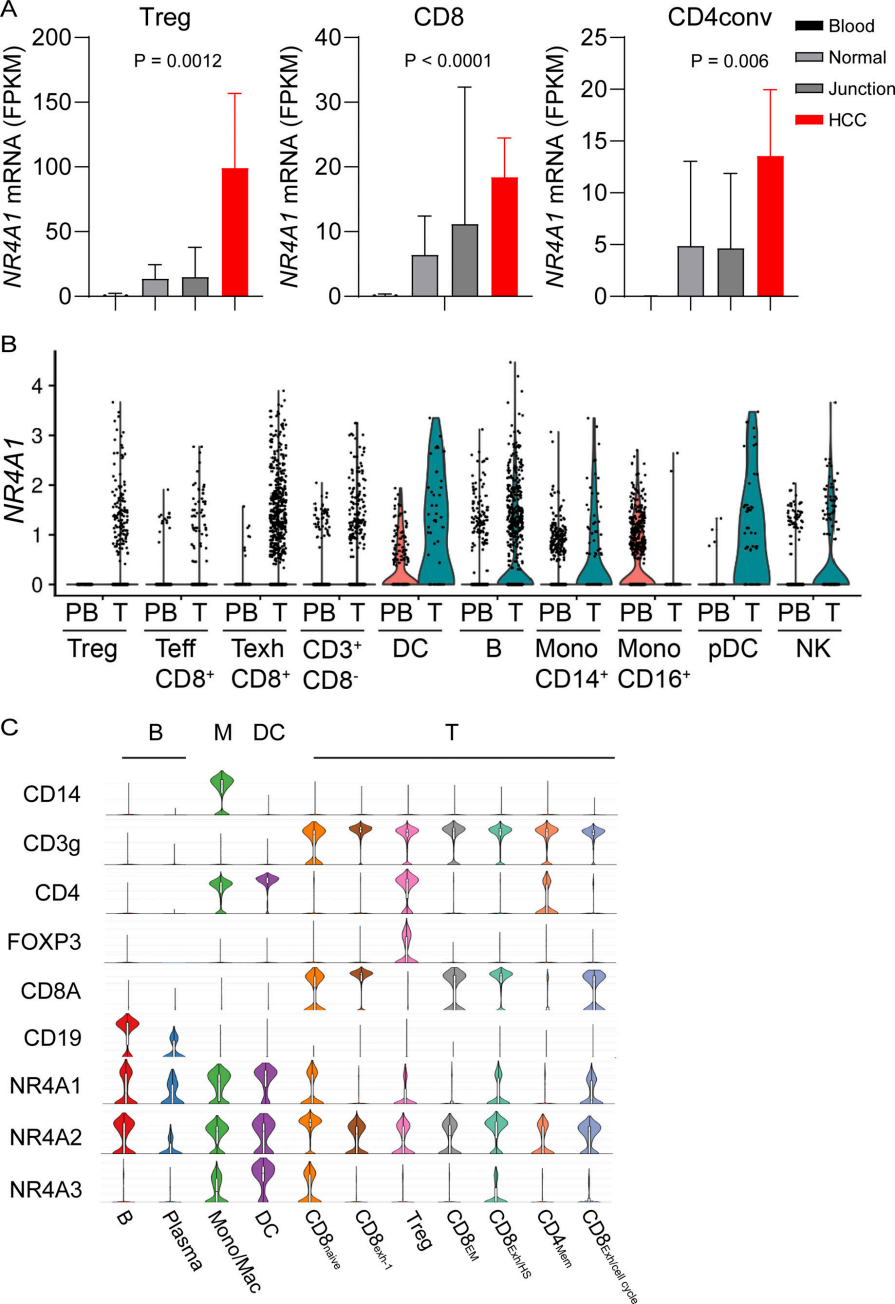
***NR4A1* is elevated in several tumor-promoting immune cells within the tumor microenvironment. (A)** Expression of *NR4A1* in T cells from blood, normal parenchyma, adjacent normal junction, or HCC (GSE98638, six patients). **(B)** Violin plots showing *NR4A1* expression in different immune cells from human peripheral blood (PB) or melanomas (T) (GEO: GSE121638, GSE158803, GSE121638). **(C)** Violin plots showing gene expression including *NR4A* family and lineage markers in human melanomas (GSE120575). FPKM, fragments per kilobase of transcript per million fragments mapped; M, monocyte.

**Figure S1.**
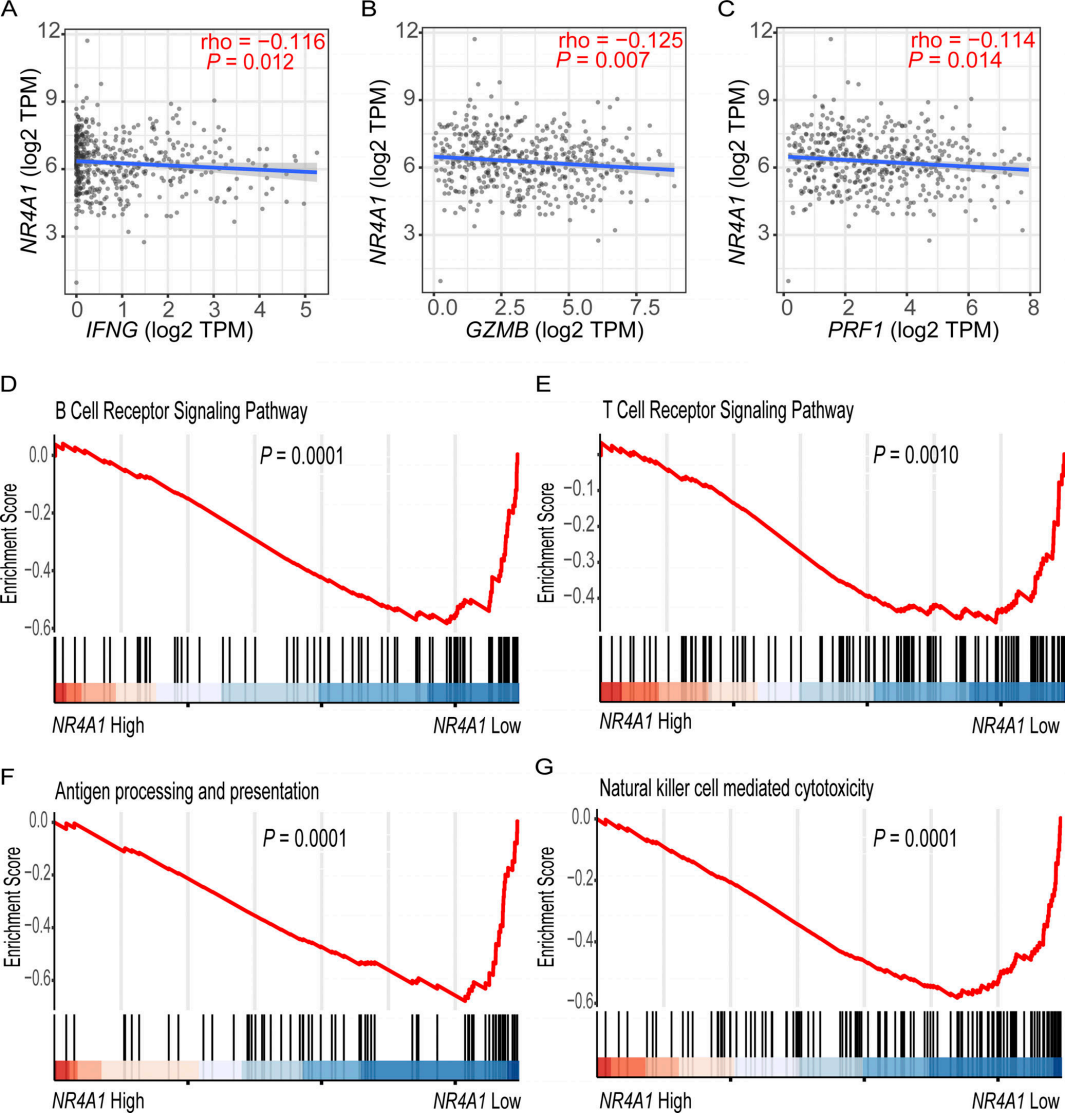
***NR4A1* expression is inversely correlated with effector molecules required for T cell activation. (A–C)** *NR4A1* expression in transcripts per million (TPM) is inversely correlated with (A) *IFNG*, (B) *GZMB*, and (C) *PRF1* gene expression in the TCGA melanoma datasets. **(D–G)** GSEA analysis showing the enrichment of immune activation pathways in the melanoma specimens with low *NR4A1* expression. The TCGA human melanoma dataset was divided into tertiles based on *NR4A1* expression. GSEA analysis was performed using the highest tertile versus the lowest tertile of *NR4A1* expression. Supplementary to Fig. 1.

We recently found that *NR4A1* is the most elevated gene in the TI-Tregs from human renal clear cell carcinoma (RCC; GEO: GSE121638) (Borcherding et al., 2021). We further analyzed *NR4A1* expression within several single-cell RNA sequencing datasets, including human hepatocellular carcinomas (HCC; GEO: GSE98638) (Zheng et al., 2017) and melanomas (GEO: GSE148190, GSE158803) (Mahuron et al., 2020; Nieto et al., 2021; Sade-Feldman et al., 2018). In HCC, we confirmed that *NR4A1* expression was significantly elevated in TI-Tregs relative to Tregs from blood or normal tissues, conventional CD4^+^ T cells, or CD8^+^ T cells (Fig. 1 A; note the difference in fragments per kilobase of transcript per million fragments mapped scales). Texh cells also had increased levels of *NR4A1*, though at much lower levels than that in TI-Tregs (Fig. 1 A). In melanoma, *NR4A1* is expressed across multiple cell types within the TME, and most TI immune cells showed elevated *NR4A1* expression relative to their blood counterparts (Fig. 1 B). *NR4A1* expression was particularly evident in B cells, monocytes or macrophages, dendritic cells (DCs), Treg cells, and Texh cells (Fig. 1 C) (Mahuron et al., 2020; Nieto et al., 2021; Sade-Feldman et al., 2018). *NR4A2* displayed a ubiquitous presence across all immune cell populations, while *NR4A3* predominantly appeared in monocytes or macrophages, DCs, and some CD8^+^ T cells (Fig. 1 C). Leveraging The Cancer Genome Atlas (TCGA) datasets, we investigated *NR4A1 *expression in melanoma patients and identified a negative correlation between *NR4A1 *and genes involved in the antitumor immune responses, including *IFNγ* (interferon γ), *GZMB* (granzyme B), and *PRF1* (perforin) (Fig. S1, A–C). Gene set enrichment analysis (GSEA) revealed that *NR4A1* expression was inversely associated with T cell receptor and B cell receptor (BCR) signaling pathways (Fig. S1, D–G). Collectively, these findings highlight the potential involvement of NR4A1 in the immune suppression of human melanomas.

**Figure 2.**
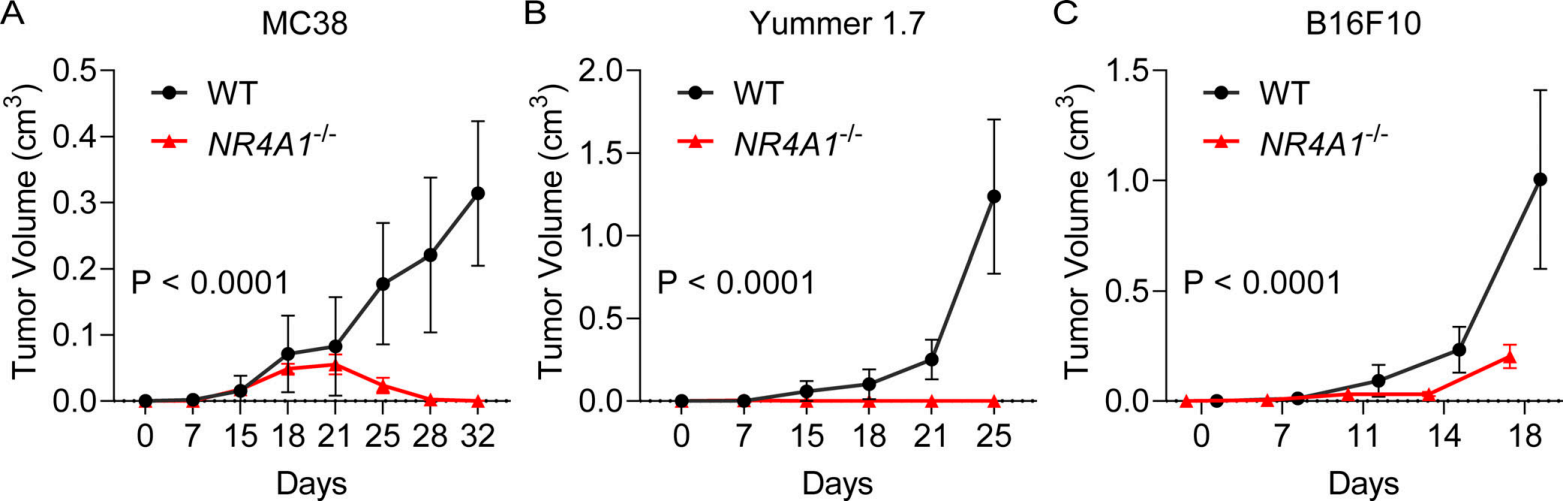
***NR4A1* deletion leads to diminished tumor growth. (A–C)** Primary tumor growth curve in littermates of WT or *NR4A1*^−/−^ mice, including (A) MC38 colon cancer (P < 0.0001, four mice per group in one experiment); (B) Yummer1.7 melanoma (P < 0.000, eight mice per group in one experiment); and (C) B16F10 melanoma (P < 0.0001, five mice per group in one experiment). Two-way ANOVA was performed for all tumor growth curves with P values indicated.

To determine whether NR4A1 plays important roles in the TME, we implanted three syngeneic tumors into wild-type (WT) or *NR4A1*^−/−^ (KO) mice, including the MC38 colon cancer model, and Yummer1.7 and B16F10, the two melanoma models. We used a minimal number of tumor cells that can produce tumors in WT mice and found that *NR4A1*^−/−^ (KO) mice exhibited a much slower tumor growth rate in all the tumor models (Fig. 2, A–C). The MC38 model showed very minimal tumor growth that peaked in the third week in the KO mice but then regressed thereafter (Fig. 2 A), suggesting that tumors were eradicated by the induction of antitumor immunity. These data support the role of NR4A1 in the TME and immune suppression and suggest that targeting NR4A1 could be a potentially promising immunotherapy for cancer.

### The design and screening of PROTACs against NR4A1

**Figure 3.**
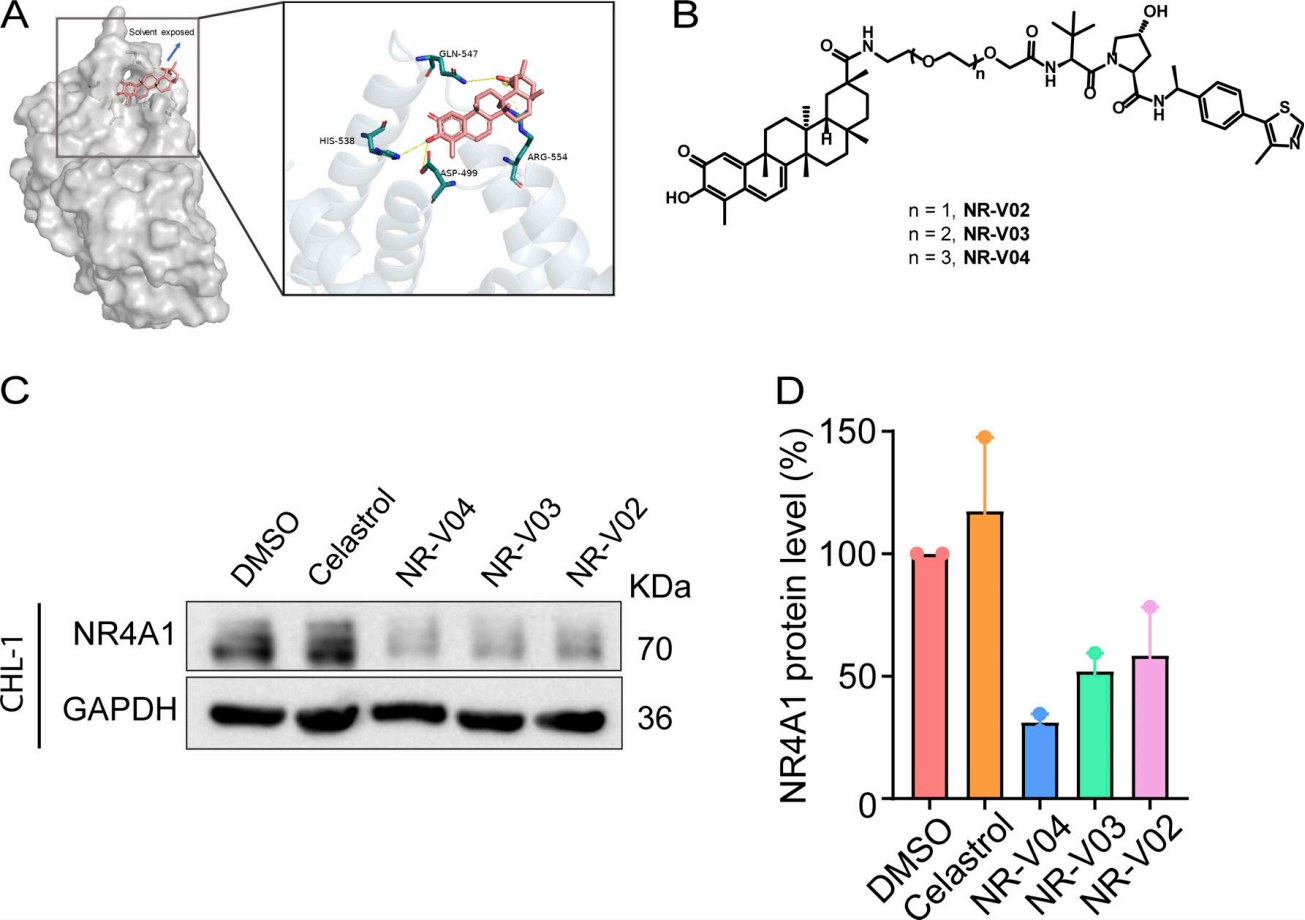
**The design and synthesis of NR4A1 PROTACs. (A)** A docking study revealed the carboxylic acid in celastrol is a potential tethering vector for PROTAC construction. **(B)** The structure of synthesized PROTACs. **(C and D)** The initial screening of NR4A1 degradation in CHL-1 cell line. CHL-1 cells were treated with 250 nM PROTACs for 16 h and the degradation was determined by (C) immunoblotting and (D) densitometry from two experiments. Source data are available for this figure: SourceData F3.

A number of NR4A1 ligands have been reported (Wu and Chen, 2018), and celastrol is one of the few that has been well characterized. Celastrol covalently binds to NR4A1 by engaging cysteine C551, and the binding affinity is within the subnanomolar range based on several biophysical assays (Chen et al., 2019b; Hu et al., 2017; Zhang et al., 2018). The structure and activity relationship study of celastrol on NR4A1 has been explored and indicated that the carboxylic acid group of celastrol is amenable to chemical modifications (Chen et al., 2019b). Therefore, we reasoned that celastrol might be a suitable warhead for PROTAC construction. We performed a molecular docking study between celastrol and the ligand binding domain (LBD) of NR4A1 and found that the carboxylic acid group is solvent exposed, which represents an ideal tethering site for linker attachment (Fig. 3 A). We utilized polyethylene glycol (PEG) linkers for the conjugation of celastrol to a VHL E3 ligase ligand via two amide bonds. As a proof-of-concept study, three PROTACs with different PEG linker lengths were synthesized (Fig. 3 B) and tested in CHL-1 human melanoma cells (Fig. 3, C and D). Celastrol treatment did not alter the protein level of NR4A1 (Fig. 3, C and D). NR-V04—which has 4-PEG linker—exhibited the highest potency in reducing the protein level of NR4A1 (Fig. 3, C and D) and was chosen for further investigation.

### NR-V04 efficiently reduces NR4A1 protein level

**Figure 4.**
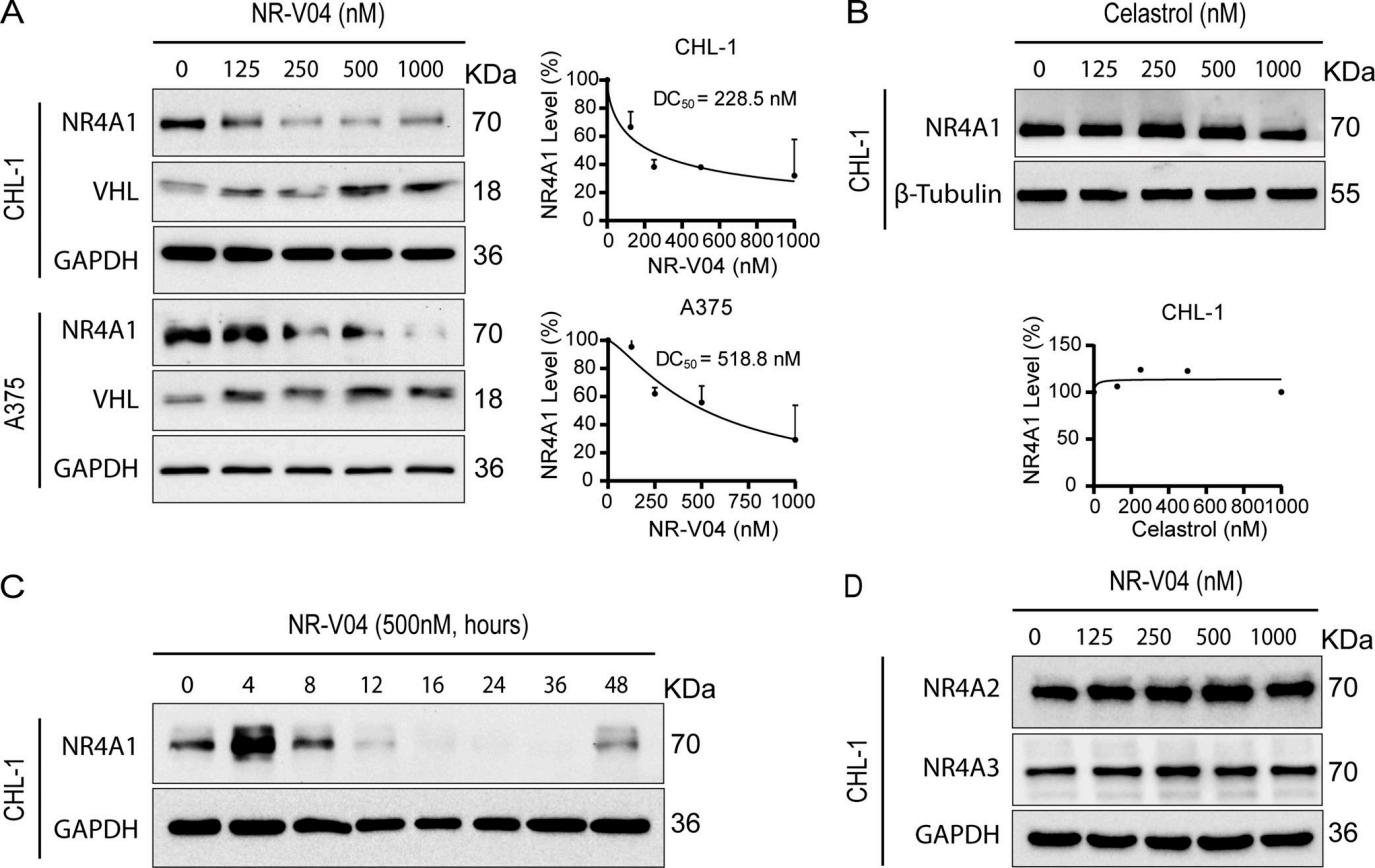
**NR-V04 induces NR4A1 degradation. (A)** NR-V04 effectively promoted the degradation of NR4A1 in two human melanoma cell lines within 16 h, CHL-1 (DC50 = 228.5 nM) and A375 (DC50 = 518.8 nM), while simultaneously stabilizing VHL protein; two experiments. **(B)** Celastrol treatment did not result in any significant change in the expression level of NR4A1 in the CHL-1 cell line within 16 h; two experiments. **(C)** Time-dependent degradation of NR4A1. CHL-1 cells were treated with 500 nM of NR-V04 at the indicated time points; two experiments. **(D)** NR-V04 did not induce the degradation of NR4A2 and NR4A3; two experiments. Source data are available for this figure: SourceData F4.

**Figure S2.**
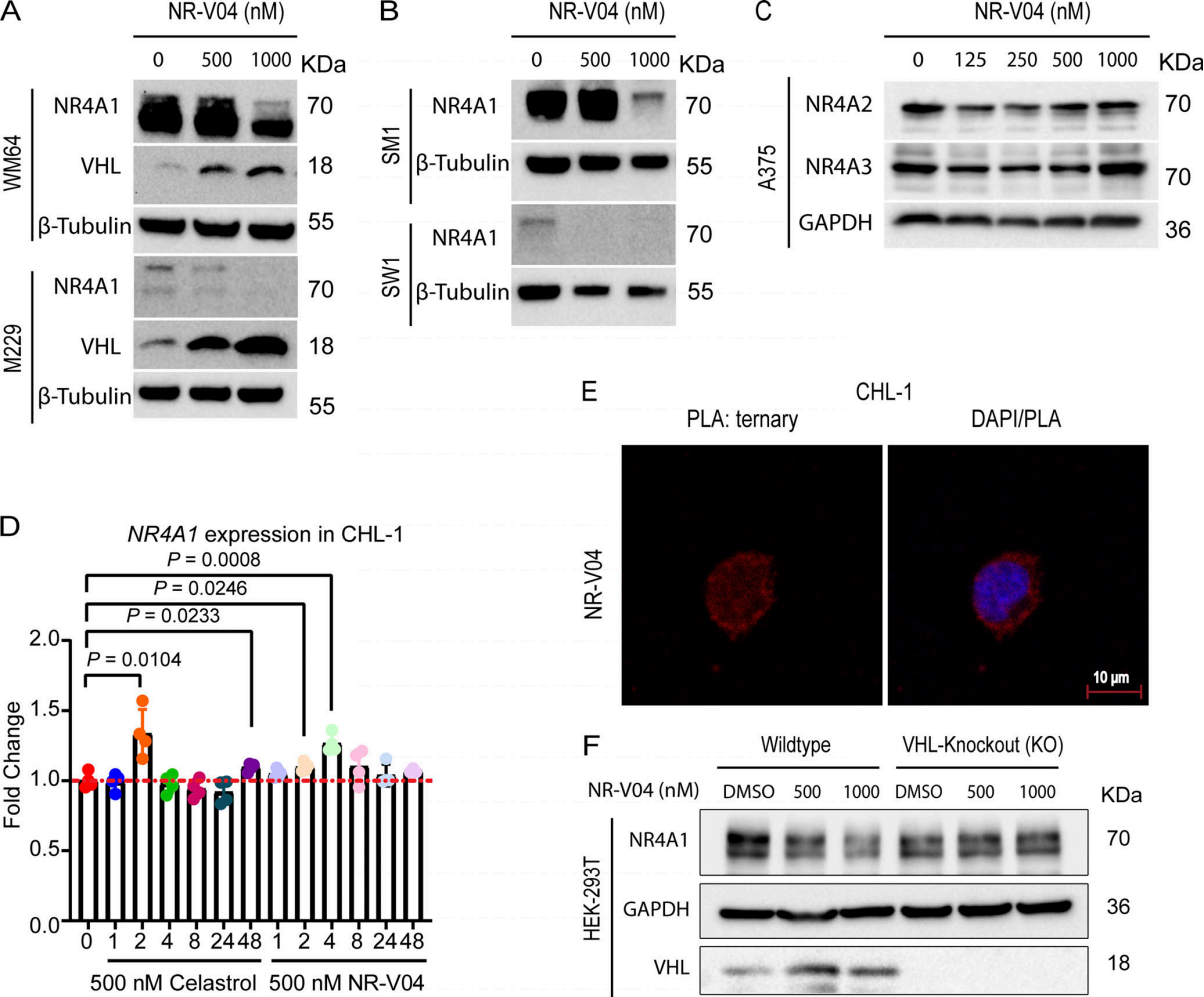
**NR-V04 induces NR4A1 degradation. (A)** NR-V04 effectively promoted the degradation of NR4A1 in two more human melanoma cell lines in 16 h, including WM164 and M229, while simultaneously stabilizing VHL expression; two experiments. **(B)** NR-V04 effectively promoted the degradation of NR4A1 in two mouse melanoma cell lines in 16 h, including SM1 and SW1; two experiments. **(C)** NR-V04 did not induce the degradation of NR4A2 and NR4A3; two experiments. **(D)** NR-V04 induces a transient elevation of *NR4A1* mRNA in short time. CHL-1 cells were treated with 500 nM of celastrol or NR-V04 at the indicated time points. RNA was prepared for reverse transcription and qPCR. *ACTB* was used as control; *N* = 4, one experiment. **(E)** Ternary complex formation was observed in CHL-1 cells with 16-h treatments of 500 nM NR-V04, rather than DMSO or 500 nM celastrol, as detected by PLA (63× magnification); two experiments. Bar represents 10 μm for both images. **(F)** NR4A1 degradation by 500 or 1,000 nM of NR-V04 for 16 h in the WT or VHL KO HEK293T cells; two experiments. A two-sided unpaired *t* test was performed, with P values indicated (P = 0.0104 between control and 2 h of celastrol treatment; P = 0.023 and 0.008, respectively, between control and 2 or 4 h of NR-V04 treatment). Supplementary to Figs. 4 and 5. Source data are available for this figure: SourceData FS2.

Following 16 h treatment, NR-V04 induced a dose-dependent decrease of NR4A1 protein in CHL-1 cells with a 50% degradation concentration (DC_50_) of 228.5 nM and in A375 melanoma cells with DC_50_ of 518.8 nM (Fig. 4 A). NR-V04 treatment also reduced NR4A1 protein level in WM164 and M229 human melanoma cells (Fig. S2 A) and SM1 and SW1—two mouse melanoma cell lines (Fig. S2 B). Celastrol did not change NR4A1 protein level (Fig. 4 B). Notably, NR-V04 treatment led to accumulation of VHL protein (Fig. 4 A and Fig. S2 A). Time course studies indicated that the efficient reduction of NR4A1 occurred between 8 and 48 h after NR-V04 treatment (Fig. 4 C). Interestingly, we observed an initial induction of NR4A1 protein after 4 h of NR-V04 treatment (Fig. 4 C) along with the timeline of increased *NR4A1* mRNA after 4 h of NR-V04 treatment (Fig. S2 D). We reasoned that this induction was mainly caused by celastrol because we observed an increase in NR4A1 mRNA levels after 2 h of celastrol treatment (Fig. S2 D). Among the three NR4A family members, NR-V04 selectively reduced the NR4A1 protein level while sparing NR4A2 and NR4A3 (Fig. 4 D and Fig. S2 C). Our data support that NR-V04 is an effective NR4A1 degrader in vitro.

### NR-V04 induces ternary complex formation and proteasome-mediated NR4A1 degradation

**Figure 5.**
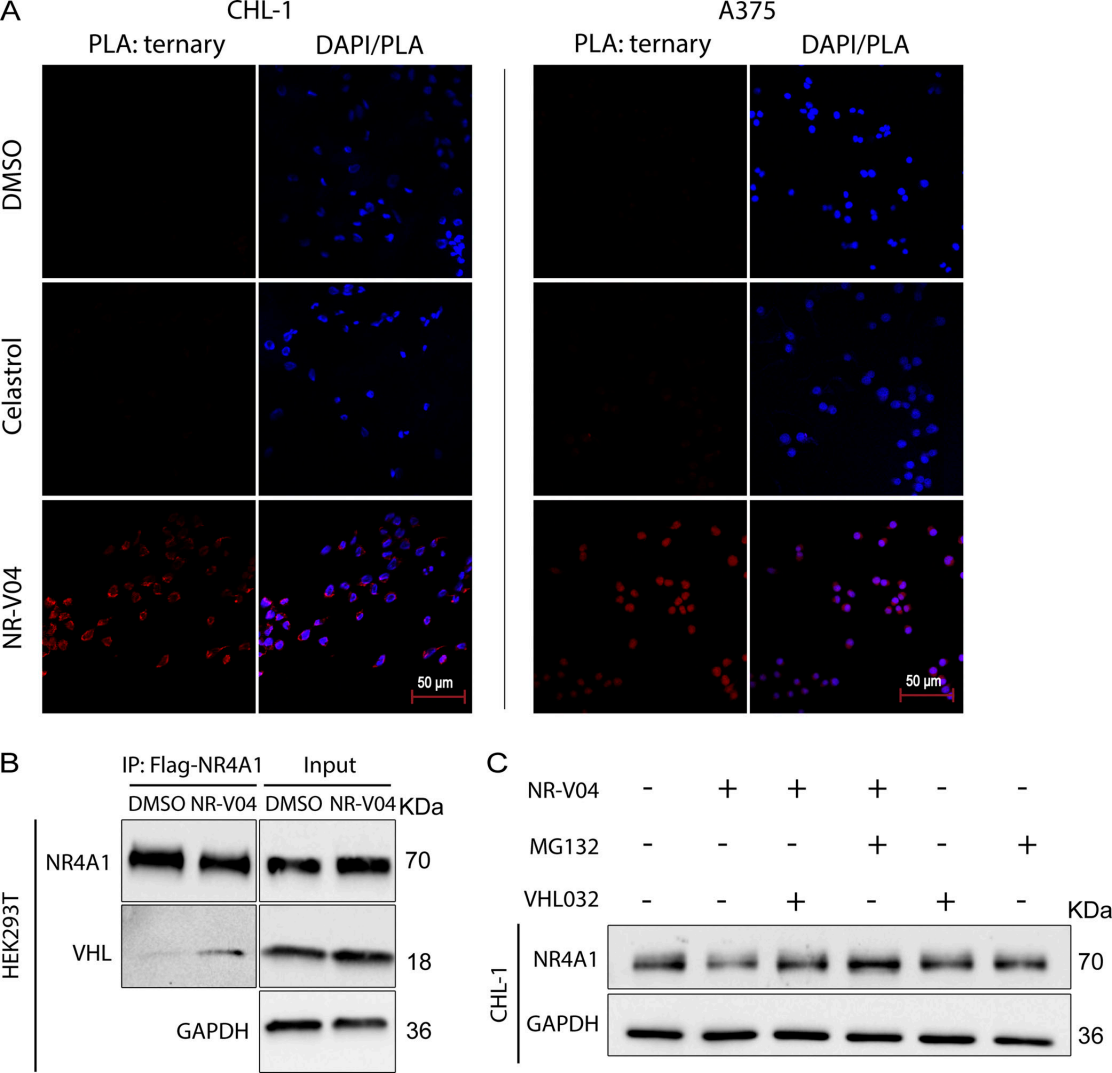
**NR-V04 induces a ternary complex formation and mediates NR4A1 degradation through the ubiquitin-proteasome system. (A)** PLA showing ternary complex formation induced by NR-V04. CHL-1 (left panels) and A375 (right panels) cells were pretreated with 0.5 μM MG132 for 10 min and then treated with DMSO, 500 nM celastrol, or 500 nM NR-V04 for 16 h. Representative images were shown for PLA assay (20× magnification); two experiments. Bar represents 50 μm for all images. **(B)** Co-IP experiment showing complex formation between NR4A1 and VHL by NR-V04 treatment. Co-IP was performed in NR4A1-Flag overexpressed HEK293T cells that were pretreated with 0.5 μM MG132 for 10 min, followed by 16-h treatment with DMSO or 500 nM NR-V04. NR4A1 was pulled down using an anti-Flag antibody conjugated to magnetic beads; two experiments. **(C)** NR-V04 induces NR4A1 degradation via VHL E3 ligase- and proteasome-dependent manner. CHL-1 cells were pretreated with 0.5 μM MG132 or 10 µM VHL 032 for 10 min, followed by 16-h treatment with DMSO or 500 nM NR-V04, three experiments. Source data are available for this figure: SourceData F5.

Ternary complex formation is a prerequisite for a PROTAC to mediate protein degradation (Békés et al., 2022). We employed proximity ligation assays (PLA) to detect localized signals only when NR4A1 and VHL were in close proximity with the presence of NR-V04. NR-V04 treatment induced strong PLA signals but celastrol or DMSO did not (Fig. 5 A and Fig. S2 E). Additionally, coimmunoprecipitation (Co-IP) using Flag-NR4A1 expressed in the HEK293T cells with and without NR-V04 treatment also supported the ternary complex formation upon NR-V04 treatment, leading to NR-V04–mediated complex formation between NR4A1 and VHL (Fig. 5 B).

We further determined the mechanism of action by which NR-V04 mediated the reduction of NR4A1 protein. MG132, a proteasome inhibitor, effectively rescued the NR4A1 protein level reduced by NR-V04 (Fig. 5 C), supporting a role in the proteasome-degradation pathway. VHL-032, the VHL ligand used for NR-V04 construction, prevented the NR-V04–induced degradation of NR4A1 (Fig. 5 C), which was further supported using the VHL-KO cells (Fig. S2 F).

### NR-V04 has good pharmacokinetic (PK) properties and shows prolonged NR4A1 degradation in vivo

**Figure 6.**
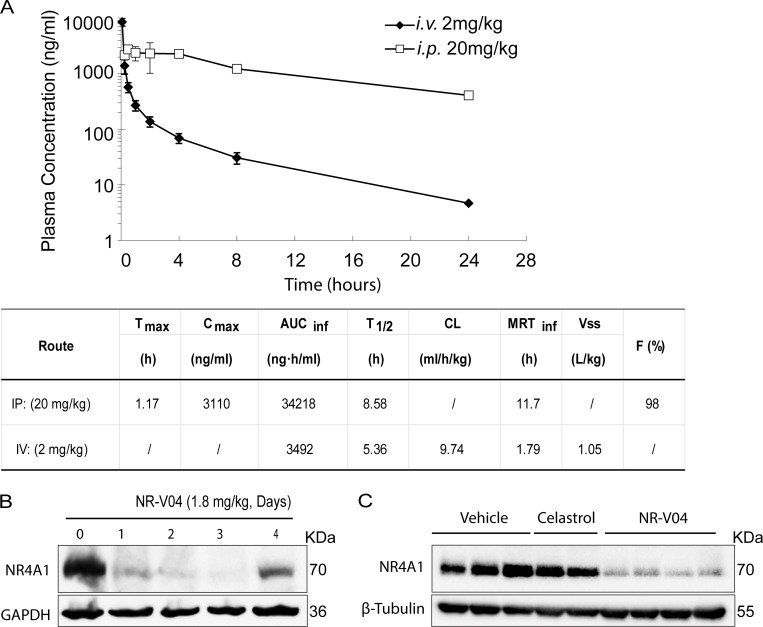
**The PK and PD study of NR-V04. (A)** PK parameters of NR-V04 in WT mice with three mice per group; one experiment. i.v. or IV, intraveneous injection; i.p. or IP, intraperitoneal injection; T_max_, time to drug peak comtration; C_max_, peak concentratin; AUC, area under the curve; T_1/2_, mean half-life; CL, clearance rate; MRT, mean residence time; Vss, steady state volume of distribution; F, fraction of bioavailability. **(B)** NR-V04 induces long-lasting degradation of NR4A1 in MC38 tumors. Mice bearing MC38 tumors were treated with two-dose administration of NR-V04 via i.p. (IP) injection at 1.8 mg/kg, and tumors were collected at indicated timepoints. Tumor lysates were analyzed by immunoblotting with each lane representing an individual tumor lysate; two experiments. **(C)** Immunoblotting showing NR4A1 degradation in MC38 tumors upon termination. MC38 tumor–bearing mice were treated with vehicle, 1.8 mg/kg NR-V04, or 0.75 mg/kg celastrol treatment (equivalent to 1.67 μmol/kg) every 4 days until experimental endpoints. Tumor tissues were collected for lysate collection and immunoblotting. Two to four biological samples per group, two experiments. Source data are available for this figure: SourceData F6.

The mouse study showed that NR-V04 had a half-life of 8.6 h via intraperitoneal injection (i.p.), 5.36 h via intravenous injection (i.v.), and near complete absorption (F = 98%) through i.p. administration with a low clearance rate (Fig. 6 A). Encouraged by the PK result, we assessed the in vivo PD properties of NR-V04. Mice bearing MC38 tumors were administered two effective doses of NR-V04 (1.8 mg/kg, determined by a pilot dose-dependent experiment), and the tumor tissues were collected and analyzed 1–4 days after the last treatment. NR-V04 treatment led to a significant degradation of NR4A1 starting from day 1 and persisting for 3 days, with a slight recovery of NR4A1 on day 4 (Fig. 6 B). We also analyzed NR4A1 protein in MC38 tumors with mice treated with vehicle, celastrol, and NR-V04 after a total of 8 doses for 4 wk in tumor-bearing mice. The data showed that NR4A1 was almost completely degraded in the NR-V04–treated group, but not in the celastrol- or vehicle-treated group (Fig. 6 C).

### NR-V04 inhibits tumor growth

**Figure 7.**
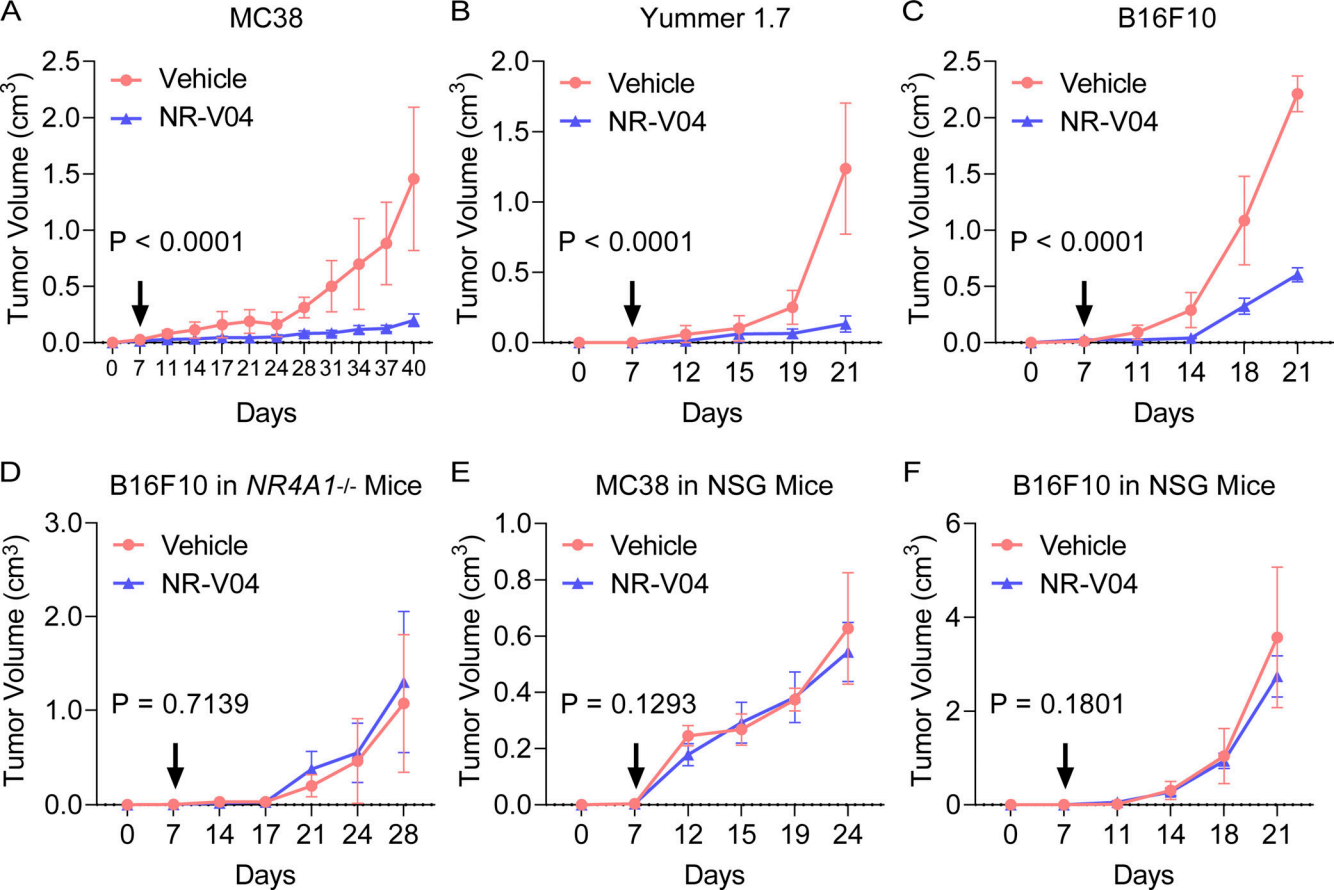
**NR-V04 exhibits antitumor effects in several tumor models. (A–F)** Tumor-bearing mice were treated with 1.8 mg/kg NR-V04 or vehicle via i.p. injection on day 7 when tumors were palpable. The treatment was repeated every 4 days until the tumors reached the endpoint size of 2 cm in diameter. **(A)** NR-V04 inhibited MC38 colon adenocarcinoma growth. P < 0.0001, four mice per group, two experiments. **(B)** NR-V04 inhibited Yummer1.7 melanoma growth. P < 0.0001, four mice per group, two experiments. **(C)** NR-V04 inhibited B16F10 melanoma growth. P < 0.0001, five mice per group, two experiments. **(D)** NR-V04 failed to inhibit B16F10 melanoma growth in *NR4A1*^−/−^ mice. P = 0.7139, five mice per group in one experiment. **(E)** NR-V04 failed to inhibit MC38 colon adenocarcinoma growth in NSG mice. P = 0.1293, five mice per group in one experiment. **(F)** NR-V04 failed to inhibit B16F10 melanoma in NSG mice. P = 0.1801, five mice per group in one experiment. Two-way ANOVA was performed for all tumor growth curves with P values indicated.

**Figure S3.**
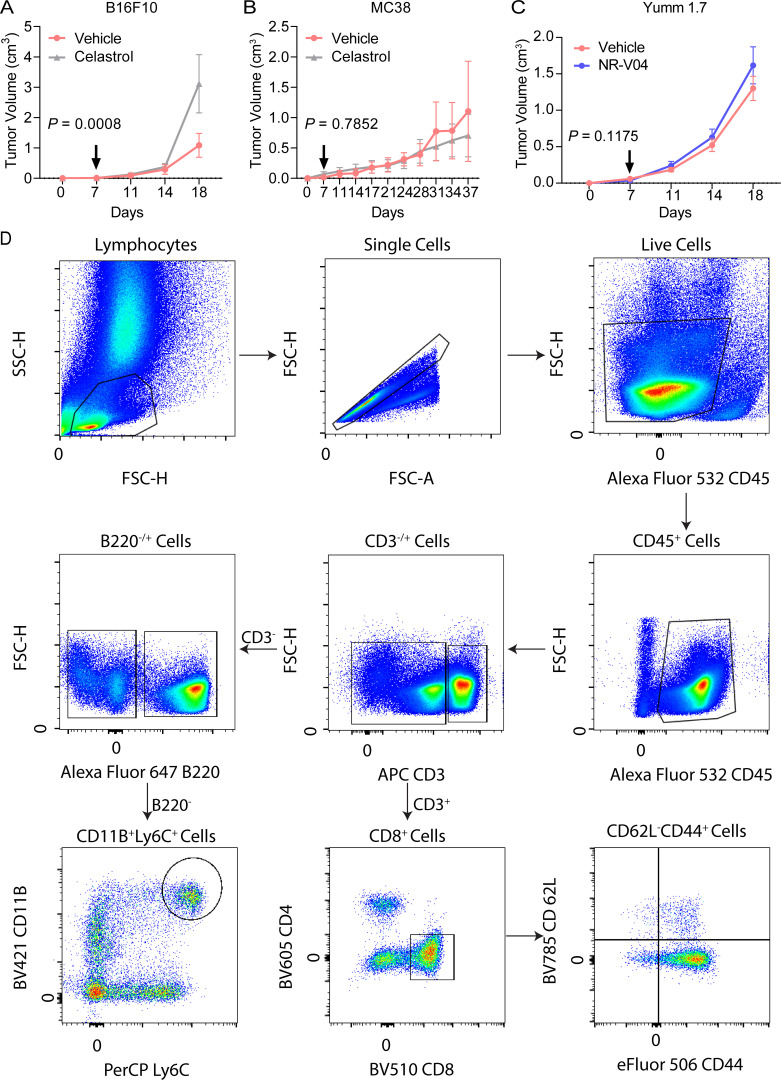
**Celastrol treatment in B16F10 and MC38 models and NR-V04 treatment in Yumm1.7 model and schematic showing gating strategy for flow cytometry. (A and B)** (A) B16F10 or (B) MC38 tumor–bearing mice were treated with vehicle or celastrol using the same treatment regimen as NR-V04 (Fig. 7). *N* = 5 for A, P = 0.0008, one experiment. *N* = 3 for B, P = 0.7852, one experiment. **(C)** Yumm1.7 (non-immunogenic Yumm cell line lineage) tumor–bearing mice were treated with vehicle or 1.8 mg/kg NR-V04. *N* = 8, P = 0.1175, one experiment. **(D)** Schematic showing gating strategy for flow cytometry of immune cell populations. Two-way ANOVA was performed for all tumor growth curves with P values indicated. Supplementary to Figs. 7 and 8.

Based on the PK and PD data, we conducted iterative optimization of NR-V04 treatment in tumor-bearing mice. Our final treatment regimen was set at a dose of 1.8 mg/kg NR-V04 or 0.75 mg/kg celastrol (equivalent to 1.67 μmol/kg) via the i.p. routes twice a week. Treatment was initiated on day 7 after inoculation of cancer cells when tumors were palpable. NR-V04 treatment effectively inhibited tumor growth in the MC38 model (Fig. 7 A) and Yummer1.7 melanoma (generated from a tumor formed in a *Braf*^V600^/*Cdkn2a*^−/−^/*Pten*^−/−^ mouse) (Fig. 7 B), as well as the commonly used B16F10 melanoma model (Fig. 7 C). In contrast, celastrol treatment resulted in either increased growth of B16F10 tumors (Fig. S3 A) or failure to inhibit tumor growth of MC38 tumors (Fig. S3 B). To validate NR4A1 as the primary molecular target of NR-V04 in the TME, we inoculated B16F10 melanoma tumors in *NR4A1*^−/−^ mice and found that NR-V04 failed to inhibit B16F10 in these mice (Fig. 7 D). *Nod*/*Scid*/*Il2rg*^−/−^ (NSG) mice lack T cells, B cells, and NK cells, and NR-V04 treatment failed to inhibit tumor growth in the NSG hosts for MC38 or B16F10 tumors (Fig. 7, E and F). Yummer1.7 is derived from Yumm1.7 (Meeth et al., 2016; Wang et al., 2017). Although they share genetic similarities, Yumm1.7 tumors have very low immunogenicity. We observed that NR-V04 showed no tumor inhibitory effect on Yumm1.7 tumors (Fig. S3 C).

### NR-V04 modulates different immune responses within the TME

**Figure 8.**
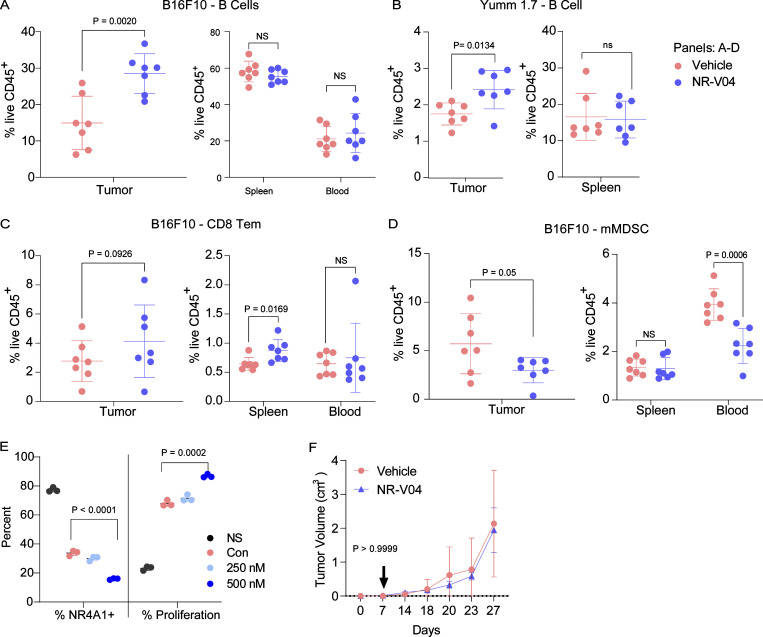
**Effect of NR-V04 on immune cells in the TME. (A–D)** Tumor-bearing mice were treated with 1.8 mg/kg NR-V04 or vehicle via i.p. injection when tumor size reached 1 cm in diameter, with two treatments on day 1 and day 4. Tumors were collected and single cells were isolated from tissues for flow cytometry analysis. **(A and B)** NR-V04 treatment increases B cell percentage in the TME, but not in spleen and blood in mice inoculated with (A) B16F10, P = 0.002 (tumor), seven tumors per group, one experiment, or (B) Yumm1.6 melanomas, P = 0.0134, seven tumors per group, one experiment. **(C)** NR-V04 treatment increased CD8 Tem cell percentage in B16F10 melanoma. P = 0.0926 (tumor) and P = 0.0169 (spleen), seven tumors per group, two experiments. **(D)** NR-V04 treatment decreased m-MDSC percentage in tumor and blood, but not in spleen in B16F10 melanoma. P = 0.05 (tumor) and P = 0.0006 (blood), seven tumors per group, two experiments. **(E)** NR4A1 depletion induces B cell proliferation. B cells isolated from spleen were labeled with Cell Trace Violet, untreated or treated with B16F10 lysis, following with the co-treatment of DMSO, 250, or 500 nM NR-V04 for 24 h. NR4A1 degradation (left) and B cell proliferation (right) were determined by flow cytometry. P < 0.0001 (left) and P = 0.0002 (right), three biological repeats, two experiments. Representative images shown in Fig. S4, E and F. **(F)** NR-V04 fails to inhibit B16F10 melanoma growth in B6.129S2-Ighmtm1Cgn/J mice deficient of mature B cells. Five tumors per group, one experiment. Two-way ANOVA was performed for (F) tumor growth curve with P value indicated. Others are shown as the mean ± SD. A two-sided unpaired *t* test was performed, with P values indicated. NS is non-significant.

**Figure S4.**
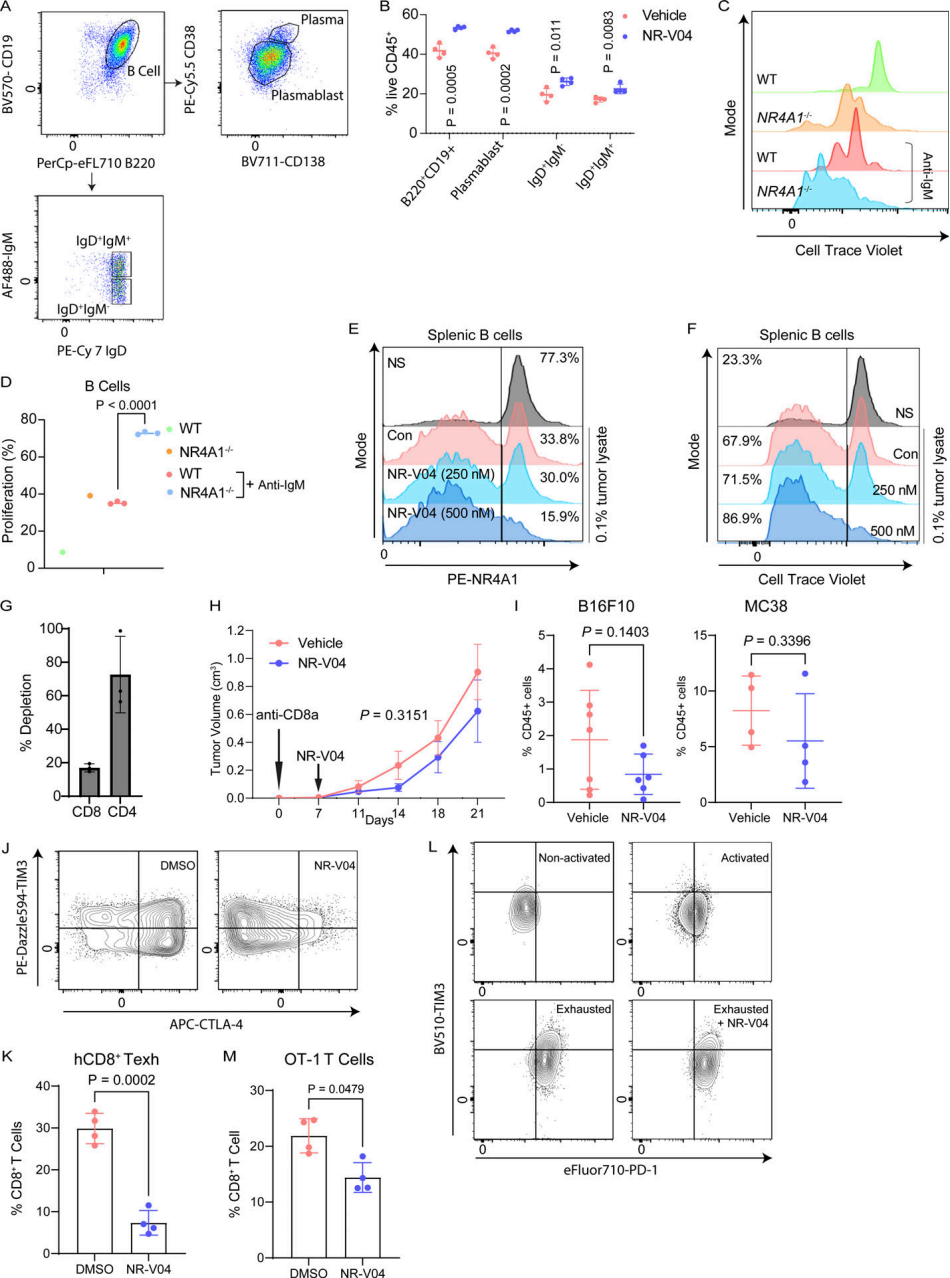
**NR-V04 regulates B and T cells. (A)** Schematic showing gating strategy for flow cytometry of B cell populations. **(B)** NR-V04 induces significant B cell proliferation in B16F10 tumors. B16F10 tumor-bearing mice were treated with vehicle and NR-V04 as in Fig. 8. *N* = 4, P = 0.0005 (B220^+^CD19^+^), P = 0.0002 (Plasmablast), P = 0.011 (IgD^+^IgM^−^), P = 0.0083 (IgD^+^IgM^+^); two experiments. **(C and D)** Splenic B cells from WT or *NR4A1*^−/−^ mice were either untreated or treated with IgM to induce B cell proliferation that was detected using flow cytometry of dilution of Cell Trace Violet; two experiments for (C) showing representative images and (D) showing all the data points. *N* = 3, P < 0.0001. **(E and F)** Splenic B cells were treated with or without B16F10 lysis stimulation in vitro and B cell proliferation was assayed similarly using flow cytometry, with or without the presence of 250 or 500 nM of NR-V04 treatment for 24 h. *N* = 3. Representative images showing (E) NR4A1 protein or (F) Cell Trace Violet dilution, determined by flow cytometry. Con, control, NS, non-stimulated by B16F10 lysate; two experiments. **(G and H)** CD8^+^ T cell depletion diminishes therapeutic responses of NR-V04. Yummer1.7 tumors were established similarly as in Fig. 7 B, following i.p. injection of either Rat IgG2a or anti-CD8 antibody (Clone: 53-6.7; BioXCell) at 100 µg/mouse, twice a week starting day 0 of tumor cell injection. NR-V04 treatment started on day 7 similarly. **(G)** Histogram showing the effective depletion of CD8 but not CD4 T cells by anti-CD8 antibody. *N* = 3. **(H)** CD8^+^ T cell depletion diminishes therapeutic responses of NR-V04 in the Yummer1.7 model, similarly treated as in Fig. 7 B. *N* = 6, P = 0.3151, one experiment. **(I)** NR-V04 did not significantly reduce CD8^+^ Texh cells in (left panel) B16F10, *N* = 7, P = 0.1403, and (right panel) MC38 tumor models, *N* = 4, P = 0.1403, from experiments shown in Fig. 7 C, and Fig. 7 A, respectively, when tumors are different in size. **(J–M)** NR-V04 reduces CD8^+^ T cell exhaustion in vitro. **(J and K)** Human primary T cells isolated from blood and cultured with anti-CD3/CD28 Dynabeads (bead/cell = 1:1) for 7 days, and then treated with 500 nM of NR-V04 for 48 h prior to harvesting and analyzed using flow cytometry. **(J)** Flow gating of human CD8^+^ Texh cells. **(K)** 500 nM of NR-V04 significantly decreased human CD8^+^ Texh cells. *N* = 4, P = 0.0002, two experiments. **(L and M)** Mouse OT-1 CD8^+^ T cells supplemented with 10 ng/ml ovalbumin daily for 7 days to induce exhaustion, following with the treatment of 500 nM NR-V04 for 48 h prior to harvesting and analyzed using flow cytometry. **(L)** Flow gating of mouse CD8^+^ OT-1 Texh cells. **(M)**. NR-V04 significantly reduced the percentage of OT-1 CD8^+^ Texh cells in CD8^+^ T cells. *N* = 4, P = 0.0479, two experiments. Two-way ANOVA was performed for all tumor growth curves with P values indicated. Others are shown as the mean ± SD. A two-sided unpaired *t* test was performed, with P values indicated. Supplementary to Fig. 8.

To better understand the immune regulatory effects of NR-V04, we designed studies to minimize the influence of tumor size on the tumor immune microenvironment. We allowed tumors to grow to 1 cm in diameter and treated tumor-bearing mice with 2 doses of NR-V04 at similar dose/time intervals (on day 1 and day 4). Tumors were collected on day 7. In the B16F10 model, NR-V04 significantly increased TI-B cells (Fig. 8 A showing B220^+^ cells from 14.7% to 30.1% and gating scheme shown in Fig. S3 D), whereas no significant change was observed in the spleen and blood. We also investigated the immune profile in the Yumm1.7 tumor mode (Meeth et al., 2016). NR-V04 treatment also resulted in a significant increase in the TI-B cells (Fig. 8 B), supporting a general mechanism of action for NR-V04 in TI-B cell induction. Furthermore, we identified the increased portion as CD38^+^CD138^−^ plasmablast-like cells and the BCR isotypes to be either IgD^+^IgM^−^ or IgD^+^IgM^+^ (Fig. S4, A and B). We also observed an increase in effector memory CD8 T cells (CD44^+^CD62L^−^ Tem) in the spleen and tumors (Fig. 8 C), as well as a decrease in monocytic myeloid-derived suppressor cells (m-MDSC) (CD11B^+^Ly6C^+^) in the tumors and blood upon NR-V04 treatment (Fig. 8 D).

To understand the mechanism of how NR4A1 regulates B cells, we purified B cells from the spleen of WT or *NR4A1*^−/−^ mice. B cells lacking NR4A1 expression exhibited increased proliferation (72.8%) compared with those from WT mice (35.2%) upon 10 μg/ml anti-IgM stimulation (Fig. S4, C and D), in agreement with published literature showing that NR4A1 is involved in limiting B cell proliferation (Tan et al., 2020). Since there is no report related to NR4A1 in TI-B cells, we treated WT B cells with B16F10 lysates to induce B cell proliferation, with or without NR-V04 treatment. NR-V04 led to NR4A1 degradation in B cells (Fig. 8 E, left, and Fig. S4 E) and significantly increased B cell proliferation (Fig. 8 E, right, and Fig. S4 F). We inoculated B16F10 into B6.129S2-*Ighm*^tm1Cgn^/J strain mice that lack mature B cells and found that NR-V04 was unable to inhibit tumor growth (Fig. 8 F), supporting that the tumor inhibitory function of NR-V04 is mediated through B cell elevation in the TME. Given the importance of CD8^+^ T cells in tumor regulation, we also depleted CD8^+^ T cells in the Yummer1.7 tumor model and treated tumor-bearing mice with NR-V04. Around 80% of CD8^+^ T cell depletion was achieved by anti-CD8 antibody when assessed at the end stage of the tumor experiment (Fig. S4 G); CD8^+^ T cell depletion desensitized tumors to NR-V04 treatment (Fig. S4 H), supporting the critical role of CD8^+^ T cells in mediating tumor suppressive effect of NR-V04. Previous studies have shown that genetic depletion of all three members of the NR4A family in CD8^+^ T cells can reverse their exhausted state (Chen et al., 2019a; Liu et al., 2019). However, we did not observe a significant decrease in CD8^+^ Texh cells in the B16F10 and MC38 mouse models in vivo following NR-V04 administration (Fig. S4 I). Curiously, we induced T cell exhaustion—assessed by surface markers—using human peripheral blood mononuclear cells (PBMC)–derived T cells (CD8^+^CTLA-4^+^Tim3^+^), as well as in mouse OT-1 CD8^+^ T cells (PD1^+^Tim3^+^); subsequently, we treated these cells with NR-V04 at a concentration of 500 nM for 48 h in vitro. NR-V04 induced a significant reduction in CD8^+^ Texh cells both in human T cells (Fig. S4, J and K) and mouse OT-1 T cells (Fig. S4, L–M). The requirement of both T and B cells indicates a potential connection between B cell induction and T cell activation, which is supported by a recent publication showing that B cells produce T cell chemokines and potentially induce CD8^+^ T cells in melanomas (Griss et al., 2019).

### NR-V04 exhibits an excellent safety profile in mice

**Figure 9.**
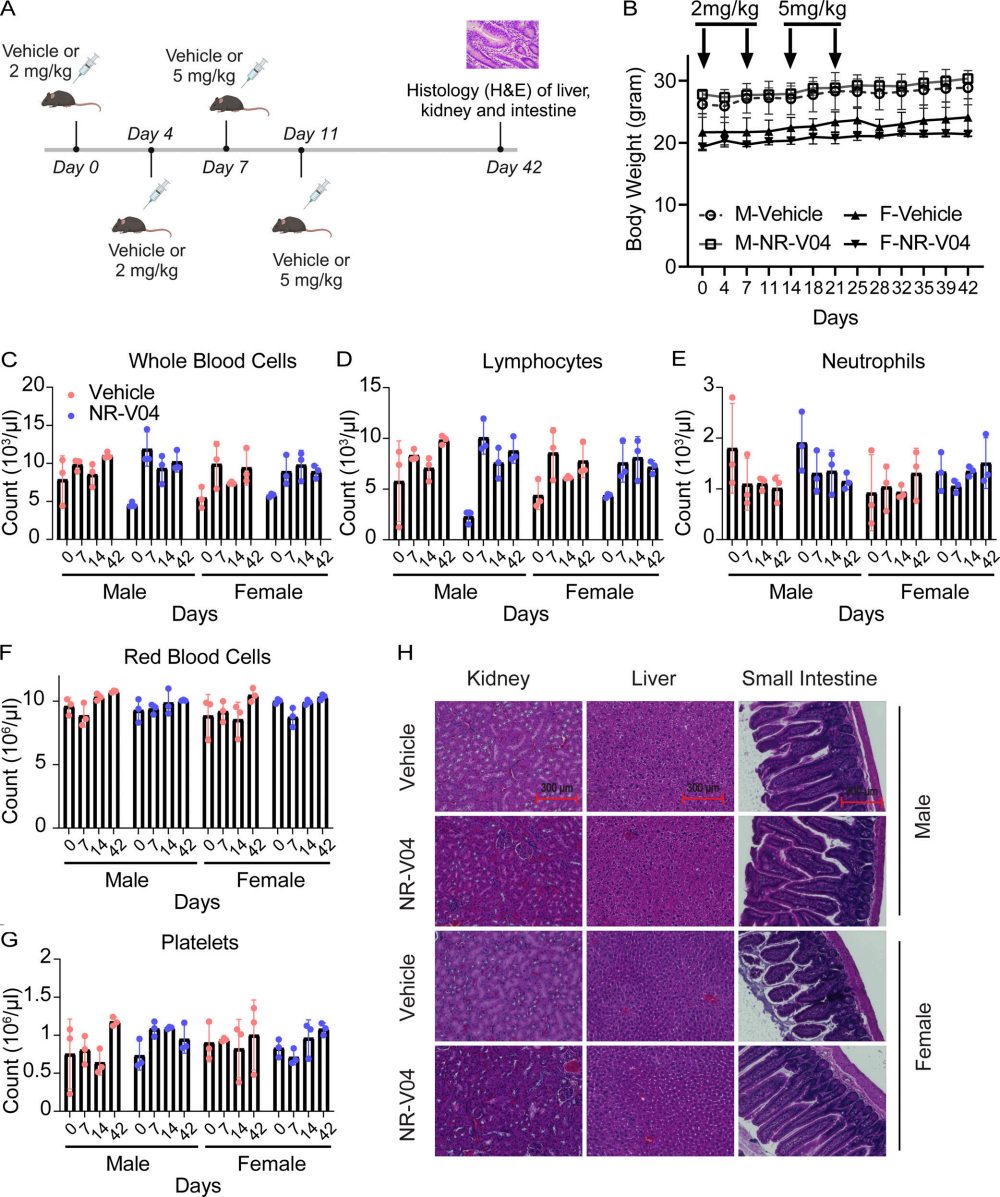
**NR-V04 has minimal toxicity. (A)** Schematic of the toxicity testing. Male and female mice were treated with two doses of 2 mg/kg NR-V04 and two doses of 5 mg/kg NR-V04 over 2 wk. Blood samples were collected on day 0, 7, 14, and 42 for hematology analysis, and body weight was measured twice per week. On day 42, all mice were euthanized, and tissues (kidney, liver, and small intestine) were harvested for H&E staining. **(B)** Mice did not experience significant weight loss with NR-V04 treatment during the 42-day period. Three biological repeats, two experiments. **(C–G)** Hematology analysis of different blood cell components after NR-V04 or vehicle treatment, including (C) whole blood cells, (D) lymphocytes, (E) neutrophils, (F) red blood cells, and (G) platelets. Three biological repeats, two experiments. **(B–G)** P > 0.05. Two-sided unpaired *t* test was performed. **(H)** NR-V04 impacts on tissue histology, including representative kidney, liver, and small intestine. Three biological repeats, one experiment. Bar represents 300 μm for all images.

**Figure S5.**
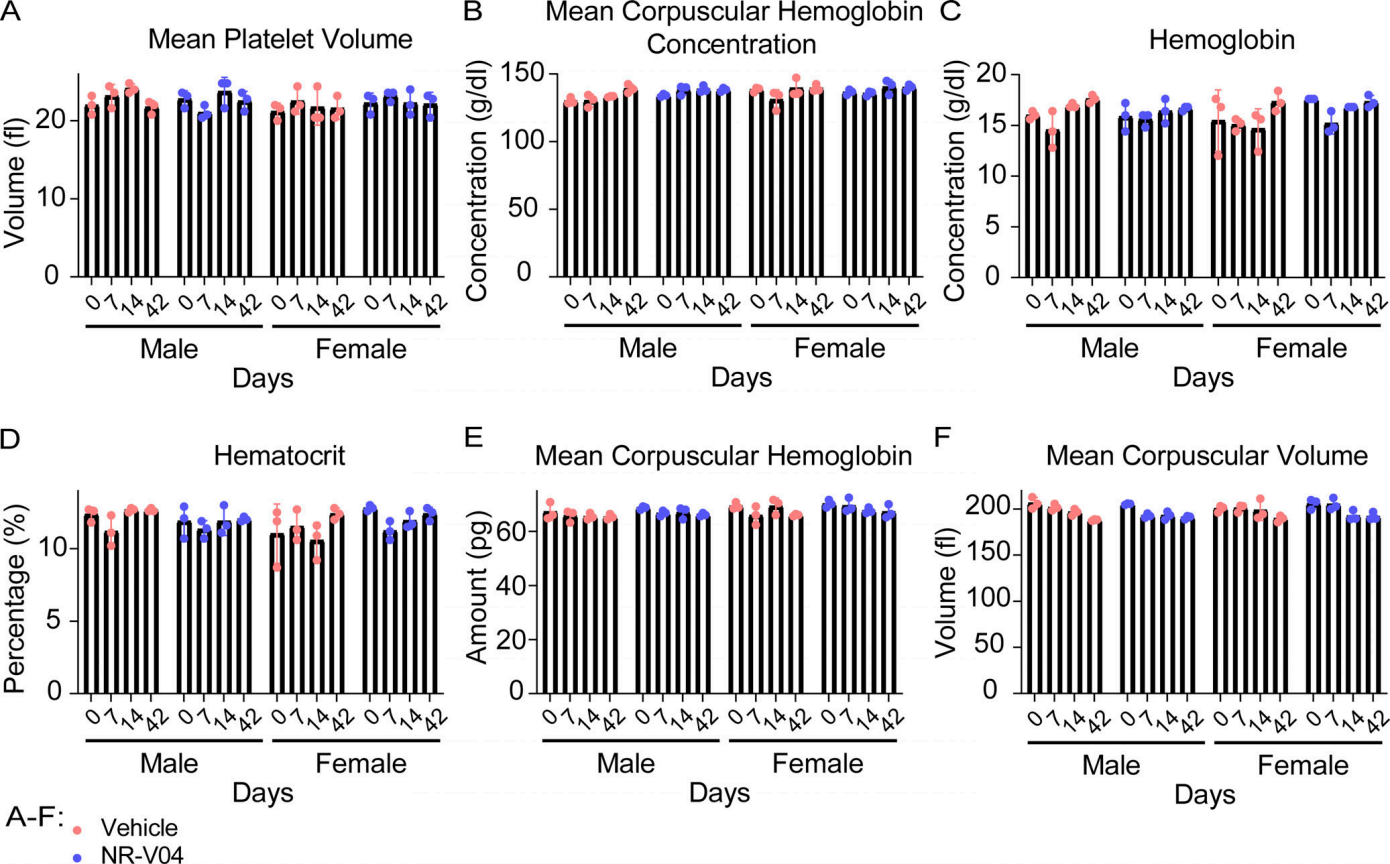
**NR-V04 has minimal toxicity. (A–F)** Hematology analysis of different blood cell components after NR-V04 or vehicle treatment. NR-V04 did not cause significant changes in the hematologic profile including (A) mean platelet volume, (B) hemoglobin, (C) mean corpuscular hemoglobin, (D) mean corpuscular hemoglobin concentration, (E) hematocrit, and (F) mean corpuscular volume. *N* = 3, P > 0.05 for A–F, one experiment. A two-sided unpaired *t* test was performed, with all P values > 0.05. Supplementary to Fig. 9.

We assessed the toxicity of NR-V04 using both male and female C57BL/6J mice with increased doses up to 5 mg/kg (Fig. 9 A). There was no significant change in body weight (Fig. 9 B). Complete blood counts were determined at different time points after NR-V04 treatment. There were no significant changes in hematological parameters, including whole blood cell count (Fig. 9 C), lymphocytes (Fig. 9 D), neutrophils (Fig. 9 E), red blood cells (Fig. 9 F), platelets (Fig. 9 G), mean platelet volume, mean corpuscular volume, hemoglobin concentration, mean corpuscular hemoglobin, hematocrit percentage, and mean corpuscular hemoglobin concentration (Fig. S5, A–F). Furthermore, we conducted a histological examination using hematoxylin and eosin (H&E) staining to evaluate the kidney, liver, and small intestine tissues in both male and female mice on day 42. Notably, NR-V04 treatment did not induce tissue damage in any of these organs (Fig. 9 H). These findings provide important insights into the potential clinical application of NR-V04 as a safe and effective immunotherapeutic agent.

## Discussion

In this study, we successfully developed NR-V04, a PROTAC that efficiently degrades NR4A1. NR-V04 has strong antitumor effects through the targeting of various cell types, such as T cells, B cells, and MDSCs. Though the cancer cell-intrinsic roles of NR4A1 have been extensively characterized in previous studies (Li et al., 2000, 2018; Smith et al., 2011), our current study strongly supports that NR4A1 degradation leads to immune activation within the TME. As such, we believe that NR4A1 is a valid target for cancer immunotherapy.

We have discussed several potential cellular targets by NR4A1 degradation, including T cells, NK cells, endothelial cells etc. (Carelock et al., 2023), but we hadn’t expected TI-B cells to be the most significantly induced cell type by NR-V04 treatment. The role of B cells within the TME is complex and has been debated depending on cancer types, B cell subpopulations, and/or the contrasting functions of TI-B cells. Most of these studies are correlative studies using human cancer specimens. For example, higher B cell numbers—often associated with the tertiary lymphoid structures—are associated with favorable clinical outcomes and improved responsiveness to immunotherapies in melanoma (Fridman et al., 2022; Sautès-Fridman et al., 2019), whereas Breg cells can mediate immunosuppression and certain B cell subpopulations promote tumor growth through proinflammatory mechanisms (De Visser et al., 2005; DeNardo et al., 2010). Here, we found that NR-V04 treatment significantly increased B cell numbers in B16F10 and Yumm1.7 mouse melanoma models where tumor sizes are controlled, and B cell deficiency abolishes the tumor inhibitory effect of NR-V04, supporting the role of B cells in NR-V04–mediated tumor suppressive effect. We do acknowledge that the frequency of B cell infiltration varies within different tumors. For example, B16F10 melanoma exhibits high B cell infiltration, accounting for >50% of total tumor-infiltrating lymphocytes, whereas Yumm1.7 shows a much lower B cell infiltration with only 2–3% of total tumor-infiltrating lymphocytes. This variation directly correlates with NR-V04’s therapeutic outcomes, implying that NR-V04 application may achieve more favorable responses in cancers with high B cell infiltrations.

NR-V04 increases CD8^+^ Tem cells in the spleens of B16F10 melanoma-bearing mice, supporting potential systematic protection of tumor cell recurrence when tumor cells are encountered with those CD8^+^ Tem cells. Within the TME, we observed a trend of increased numbers of CD8^+^ Tem cells in vivo. CD8 T cell depletion diminished the therapeutic response of NR-V04 (Fig. S4, G and H), supporting a role for CD8^+^ T cells in the therapeutic effect of NR-V04. B cells have the potential to stimulate T cell activation within the TME (Sharonov et al., 2020), and in melanoma they produce T cell chemokines to potentially recruit T cells into the TME (Griss et al., 2019). We did not detect significant changes in CD8^+^ Texh cells, TI-Tregs, or -NK cells upon NR-V04 treatment while controlling for tumor size, but certainly acknowledge that NR4A1 and its family members are important for these TI lymphocyte populations. We did observe that NR-V04 was able to reduce the CD8^+^ Texh cells in both human and mouse models in vitro; however, we only observed a trend but no statistical significance in vivo. The in vivo results likely reflect the compensatory functions of NR4A2 or NR4A3 in T cell exhaustion or other Texh-related changes such as altered cytokine production that cannot be revealed via staining of surface exhaustion markers. We did notice some interesting changes in our initial experiments using tumors with different sizes from Fig. 7, A–C, but did not include those data since the tumor sizes are drastically different, which could be a confounding factor for assessing TI immune cells.

NR-V04’s impact on the TME extends beyond regulating T and B cells. NR-V04 decreases m-MDSCs in tumors and blood. m-MDSCs are known to suppress the immune response, including T and B cell functions (Li et al., 2021; Wang et al., 2018). Furthermore, B cell activation results in immune complex formation that attracts proinflammatory cytokines produced by m-MDSCs (Fridman et al., 2022; Rosser et al., 2014). Thus, NR-V04’s action on m-MDSCs can potentially alleviate immune suppression, thereby enhancing T and/or B cell–mediated antitumor effects.

The PK-PD decoupled, long-lasting degradation effect of NR-V04 in vivo (Fig. 6 B) is expected for a PROTAC molecule, but could also suggest that NR-V04 can specifically accumulate in the tumors, a favorable feature for drug development that warrants reasonable lower effective doses and longer treatment intervals. The warhead used in NR-V04 is celastrol, which has been associated with several adverse effects such as hepatotoxicity (Jin et al., 2019; Sun et al., 2014), hematopoietic system toxicity (Lipsky and Tao, 1997), nephrotoxicity (Wu et al., 2018), weight loss, and negative impact on metabolic and cardiovascular functions (Feng et al., 2019; Liu et al., 2015; Saito et al., 2019). However, with the same treatment regimen, NR-V04 exhibits an excellent safety profile in vivo. This safety advantage could be attributed to NR-V04’s superior specificity as a PROTAC that targets NR4A1 rather than a collection of other known celastrol targets, which reduces off-target effects that could lead to toxicity in patients. One caveat related to the NR-V04 is the usage of VHL, which is a well-established tumor suppressor gene (Gossage et al., 2015) and very commonly mutated in human cancers such as RCCs. We have been actively developing other E3-recruiting NR4A1 PROTACs, but at the current stage, none of those different PROTACs exhibit better tumor suppression and safety profile than NR-V04.

## Materials and methods

### Chemistry

Dimethylformamide (DMF) and dichloromethane (DCM) were obtained via a solvent purification system by filtering through two columns packed with activated alumina and a 4 Å molecular sieve, respectively. Water was purified with a Milli-Q Simplicity 185 Water Purification System (Merck Millipore). All other chemicals and solvents obtained from commercial suppliers were used without further purification. Flash chromatography was performed using silica gel (230–400 mesh) as the stationary phase. Reaction progress was monitored by thin-layer chromatography (silica-coated glass plates) and visualized by 256 and 365 nm UV light, and/or by liquid chromatography–mass spectrometry (LC-MS). ^1^H nuclear magnetic resonance (NMR) spectra were recorded in CDCl_3_ or CD_3_OD at 600 MHz, and ^13^C NMR spectra were recorded at 151 MHz using a Bruker DRX NMR spectrometer. Chemical shifts δ are given in ppm using tetramethylsilane as an internal standard. Multiplicities of NMR signals are designated as singlet (s), doublet (d), doublet of doublets (dd), triplet (t), quartet, a triplet of doublets, a doublet of triplets, and multiplet (m). All final compounds for biological testing were of ≥95.0% purity as analyzed by LC-MS, performed on an Advion AVANT LC system with the expression Compact Mass Spectrometer using a Thermo Accucore Vanquish C18+ Ultra High-Performance Liquid Chromatography (UHPLC) Column (1.5 µm, 50 × 2.1 mm) at 40°C. Gradient elution was used for UHPLC with a mobile phase of acetonitrile and water containing 0.1% formic acid. High-resolution mass spectra were recorded on an Agilent 6230 time-of-flight mass spectrometer.

Intermediates **1a** to **3a** were synthesized according to a previously reported procedure (Xiao et al., 2020). Briefly, to a solution of VHL-amine HCl salt (1.0 equiv) and corresponding acid-terminated linkers (1.0 equiv) in 5 ml DCM was added hexafluorophosphate azabenzotriazole tetramethyl uronium (HATU) (1.1 equiv) and N, N-Diisopropylethylamine (DIPEA) (5.0 equiv), and the reaction was stirred at room temperature overnight. After extraction with ethyl acetate and brine, the residue was concentrated and purified by silica gel column chromatography to yield **1a** to **3a**.

Tert-butyl (2-(2-(2-(((S)-1-((2S,4R)-4-hydroxy-2-(((S)-1-(4-(4-methylthiazol-5-yl)phenyl)ethyl)carbamoyl)pyrrolidin-1-yl)-3,3-dimethyl-1-oxobutan-2-yl)amino)-2-oxoethoxy)ethoxy)ethyl)carbamate (**1a**) was obtained as a colorless oil with the 93% yield: ^1^H NMR (600 MHz, Chloroform-*d*) δ 8.67 (s, 1H), 7.57–7.35 (m, 5H), 7.33–7.27 (m, 1H), 5.20–5.03 (m, 1H), 4.79–4.50 (m, 3H), 4.17–3.80 (m, 3H), 3.69–3.49 (m, 7H), 3.45–3.28 (m, 2H), 2.57–2.51 (m, 4H), 2.22–1.99 (m, 1H), 1.59–1.48 (m, 12H), 1.05 (s, 9H). Electrospray Ionization (ESI) [M+H]^+^ = 690.4.

Tert-butyl ((S)-13-((2S,4R)-4-hydroxy-2-(((S)-1-(4-(4-methylthiazol-5-yl)phenyl)ethyl)carbamoyl)pyrrolidine-1-carbonyl)-14,14-dimethyl-11-oxo-3,6,9-trioxa-12-azapentadecyl)carbamate (**2a**) was obtained as a colorless oil with the 91% yield: ^1^H NMR (600 MHz, Chloroform-*d*) δ 8.69 (s, 1H), 7.54–7.46 (m, 1H), 7.43–7.33 (m, 5H), 5.20 (s, 1H), 5.11–5.01 (m, 1H), 4.71 (t, *J* = 7.8 Hz, 1H), 4.63–4.53 (m, 1H), 4.55–4.46 (m, 1H), 4.08–3.93 (m, 3H), 3.73–3.61 (m, 9H), 3.54 (t, *J* = 5.4 Hz, 2H), 3.36–3.26 (m, 2H), 2.52 (s, 3H), 2.42–2.34 (m, 1H), 2.15–2.07 (m, 1H), 1.48 (d, *J* = 7.0 Hz, 3H), 1.43 (s, 9H), 1.06 (s, 9H). ESI [M+H]^+^ = 733.3.

Tert-butyl ((S)-16-((2S,4R)-4-hydroxy-2-(((S)-1-(4-(4-methylthiazol-5-yl)phenyl)ethyl)carbamoyl)pyrrolidine-1-carbonyl)-17,17-dimethyl-14-oxo-3,6,9,12-tetraoxa-15-azaoctadecyl)carbamate (**3a**) was obtained as colorless oil with the 91% yield: ^1^H NMR (600 MHz, Chloroform-*d*) δ 8.68 (s, 1H), 7.46–7.41 (m, 1H), 7.41–7.35 (m, 4H), 7.26–7.20 (m, 1H), 5.27–5.16 (m, 1H), 5.10–5.02 (m, 1H), 4.70 (t, *J* = 7.9 Hz, 1H), 4.59 (d, *J* = 8.7 Hz, 1H), 4.51 (s, 1H), 4.06 (s, 2H), 3.95 (d, *J* = 11.2 Hz, 1H), 3.80 (s, 1H), 3.71–3.59 (m, 12H), 3.56 (t, *J* = 5.2 Hz, 2H), 3.36–3.26 (m, 2H), 2.83–2.75 (m, 1H), 2.52 (s, 3H), 2.40–2.31 (m, 1H), 2.15–2.07 (m, 1H), 1.50–1.39 (m, 12H), 1.05 (s, 9H). ESI [M+H]^+^ = 778.4.

General procedure for the synthesis of PROTACs is as follows: **1a**, **2a**, or **3a** (1.0 equiv) was dissolved in 5 ml DCM/TFA (2:1) and stirred at room temperature for 6 h. The resulting mixture was concentrated under vacuum and redissolved in DMF, and celastrol was added followed by DIPEA (5.0 equiv) and HATU (1.1 equiv). The reaction mixture was then stirred at room temperature overnight. Water (20 ml) and EtOAc (40 ml) were added. The organic phase was washed with brine (10 ml), dried over Na_2_SO_4_, filtered, and concentrated. The residue was purified by silica gel column chromatography to yield NR-V02, NR-V03, and NR-V04, respectively.

NR-V02 was obtained as red solid (14 mg, 50% yield). ^1^H NMR (600 MHz, Chloroform-*d*) δ 8.67 (s, 1H), 7.40–7.33 (m, 6H), 7.08 (s, 1H), 7.03 (dd, *J* = 7.1, 1.4 Hz, 1H), 6.53 (s, 1H), 6.44–6.40 (m, 1H), 6.34 (d, *J* = 7.4 Hz, 1H), 5.11–5.07 (m, 1H), 4.72 (t, *J* = 8.1 Hz, 1H), 4.56 (d, *J* = 8.6 Hz, 1H), 4.52 (s, 1H), 4.12–3.96 (m, 3H), 3.76–3.13 (m, 12H), 2.52 (s, 3H), 2.47–2.39 (m, 2H), 2.20 (s, 3H), 2.17–1.76 (m, 7H), 1.69–1.41 (m, 11H), 1.24 (s, 3H), 1.16–0.97 (m, 16H), 0.61 (s, 3H). ^13^C NMR (151 MHz, CDCl_3_) δ 178.52, 178.35, 171.51, 171.10, 170.68, 169.89, 165.08, 150.32, 148.47, 146.11, 143.22, 134.68, 131.61, 130.84, 129.55, 127.28, 126.42, 119.48, 118.11, 117.71, 70.84, 70.14, 70.07, 58.69, 57.46, 56.77, 48.88, 47.40, 46.33, 45.11, 44.36, 43.14, 40.38, 39.39, 39.35, 38.19, 36.35, 35.94, 35.27, 34.87, 33.83, 33.50, 31.62, 31.05, 30.76, 29.96, 29.71, 29.38, 28.64, 26.53, 26.45, 26.39, 22.23, 21.68, 18.40, 16.11, 10.30, 8.72. MS m/z: [M+H]^+^ calculated for C_58_H_80_N_5_O_9_S_1_^+^: 1022.5671, found 1022.5680.

NR-V03 was obtained as red solid (12 mg, 51% yield). ^1^H NMR (600 MHz, Chloroform-*d*) δ 8.67 (s, 1H), 7.48–7.35 (m, 5H), 7.24 (d, *J* = 8.5 Hz, 1H), 7.11–7.05 (m, 1H), 7.02 (d, *J* = 7.0, 1.4 Hz, 1H), 6.53–6.50 (m, 1H), 6.37–6.30 (m, 2H), 5.14–5.05 (m, 1H), 4.72 (t, *J* = 7.9 Hz, 1H), 4.57 (d, *J* = 8.7 Hz, 1H), 4.52 (s, 1H), 4.09–4.00 (m, 3H), 3.87–2.98 (m, 16H), 2.53 (s, 3H), 2.50–2.39 (m, 2H), 2.20 (s, 3H), 2.15–1.81 (m, 7H), 1.69–1.36 (m, 11H), 1.25 (s, 3H), 1.12 (d, *J* = 16.9 Hz, 6H), 1.05 (s, 9H), 1.02–0.96 (m, 1H), 0.62 (s, 3H). ^13^C NMR (151 MHz, CDCl_3_) δ 178.46, 178.31, 171.44, 170.90, 170.53, 169.90, 165.02, 150.29, 148.48, 146.09, 143.31, 134.51, 131.63, 130.82, 129.54, 127.33, 126.45, 119.45, 118.10, 117.55, 70.67, 70.34, 70.31, 70.12, 69.87, 67.99, 58.60, 57.33, 56.72, 48.84, 47.40, 45.11, 44.37, 43.09, 40.29, 39.34, 39.29, 38.25, 36.35, 35.89, 35.20, 34.92, 33.79, 33.54, 31.62, 31.09, 30.78, 29.99, 29.71, 29.36, 28.68, 26.51, 26.40, 25.62, 22.23, 21.70, 18.38, 16.12, 10.29, 8.73. MS (ESI); m/z: [M+H]^+^ calculated for C_60_H_84_N_5_O_1_S_1_^+^: 1066.5933, found 1066.5933.

NR-V04 was obtained as red solid (24 mg, 55% yield). ^1^H NMR (600 MHz, Chloroform-*d*) δ 8.67 (s, 1H), 7.43–7.35 (m, 5H), 7.17 (s, 1H), 7.04 (d, *J* = 7.0 Hz, 1H), 6.53 (s, 1H), 6.45 (s, 1H), 6.34 (d, *J* = 7.2 Hz, 1H), 5.12–5.05 (m, 1H), 4.68 (t, *J* = 8.0 Hz, 1H), 4.57 (d, *J* = 8.9 Hz, 1H), 4.51 (s, 1H), 4.09–3.92 (m, 3H), 3.73–3.15 (m, 17H), 2.53 (s, 3H), 2.48–2.38 (m, 2H), 2.21 (s, 3H), 2.16–1.80 (m, 12H), 1.69–1.42 (m, 13H), 1.25 (s, 3H), 1.12 (d, *J* = 14.7 Hz, 6H), 1.06–0.92 (m, 10H). ^13^C NMR (151 MHz, CDCl_3_) δ 179.35, 178.35, 171.61, 171.14, 170.37, 170.25, 165.37, 150.30, 148.41, 146.17, 143.34, 135.18, 131.68, 130.73, 129.52, 127.21, 126.53, 119.36, 118.24, 118.06, 70.89, 70.04, 69.89, 69.66, 69.51, 59.04, 57.30, 56.84, 48.89, 45.20, 44.29, 43.20, 40.44, 39.31, 39.12, 38.19, 36.31, 36.23, 35.51, 34.65, 33.83, 33.55, 31.57, 30.79, 29.75, 29.27, 28.65, 26.36, 22.09, 21.61, 18.30, 16.11, 10.30. MS (ESI); m/z: [M+H]^+^ calculated for C_62_H_88_N_5_O_11_S_1_^+^: 1110.6196, found 1110.6199.

### Docking study

The crystal structure of human NR4A1 LBD domain (PDB: 4KZI) was utilized for the modeling. The protein preparation workflow in Maestro 13.3 (Schrödinger) was used to preprocess and optimize H-bond assignment, minimize energy, and delete water. Celastrol was prepared by the LigPrep module with the OPLS4 force field. Grid box was generated centroid of Cys551 with 20 Å. Standard precision mode was selected with default parameters. The final disposition with low-energy conformation was selected. The result was visualized by PyMOL software.

### Single-cell RNA analysis

We utilized multiple datasets from GEO (GSE148190, GSE158803, GSE12163, GSE120575) and the human TCGA melanoma data cohort for analysis. Data visualization and analysis were conducted using Single Cell Portal (https://singlecell.broadinstitute.org/single_cell; Broad Institute), Seurat R package (V2.3.4), and GSEA. The code used in our analysis is available on GitHub (https://github.com/Levy0803/NR4A1).

### Cell lines and cell culture

Human melanoma cell lines: A375 and CHL-1 (gifts from Dr. D. Zhou, the University of Texas Health Science Center, San Antonio, TX, USA) were cultured in Dulbecco’s Modified Eagles’ Medium (DMEM, D5796; Sigma-Aldrich), WM164 and M229 (gifts from Dr. K.S.M. Smalley’s lab) were cultured in RPMI-1640 (R8758; Sigma-Aldrich). Mouse melanoma cell lines: B16F10 (CRL-6475, ATCC) was cultured in DMEM, SW1 and SM1 (gift from Dr. K.S.M. Smalley, Moffitt Cancer Center, Tampa, FL, USA) were cultured in RPMI. Human epithelial kidney cell lines: HEK293T (293T, CRL-3216; ATCC) and Vhl-KO-293T (gift from Dr. D. Zhou, the University of Texas Health Science Center, San Antonio, TX, USA) were cultured in DMEM. Mouse colon cancer cell lines: MC38 (ENH204-FP; Kerafast Inc.) was cultured in DMEM supplemented with 1 mM glutamine, 0.1 M non-essential amino acids (11140050; Thermo Fisher Scientific), 1 mM sodium pyruvate (11360070; Thermo Fisher Scientific), and 10 mM HEPES (15630080; Thermo Fisher Scientific). All cell culturing media were supplemented with 10% fetal bovine serum (FBS, F2442; Sigma-Aldrich), 100 U/ml penicillin, and 100 µg/ml streptomycin (P4333; Sigma-Aldrich).

### Immunoblotting

Samples were lysed using radioimmunoprecipitation assay buffer (RIPA) lysis buffer (150 mM NaCl, 5 mM EDTA, 50 mM Tris pH 8.0, 0.5% sodium deoxycholate, 1% NP-40, 0.1% SDS) supplemented with 1 mM dithiothreitol and protease inhibitors. The lysates were separated by SDS-PAGE and analyzed using standard western blotting procedures (Khan et al., 2019). Antibodies: human NR4A1 (ab153914, 1:1,000; Abcam), mouse NR4A1 (14-5965-82, 1:1,000; Invitrogen), NR4A2 (AV38753, 1:1,000; Sigma-Aldrich), NR4A3 (TA804893, 1: 1,000; Thermo Fisher Scientific), VHL (68547, 1:1,000; Cell Signaling), β-actin (13E5, 1:5,000; Cell Signaling), β-tubulin (9F3, 1: 5,000; Cell Signaling), and GAPDH (D16H11, 1: 5,000; Cell Signaling) were employed for protein detection.

### Cell transfection

HEK293T cells were transfected with 3 µg Flag-NR4A1 (HG17699-CF; SinoBiological) for 36 h in Opti-MEM (31985062; Thermo Fisher Scientific) medium with 10 µl GeneTran III reagent (GT2211; Biomiga).

### Co-IP

HEK293T cells were transfected with Flag-NR4A1 for 36 h, following with treatment of either DMSO or 500 nM NR4A1 for 16 h, together with 500 nM MG132 included to prevent protein degradation. Proteins were extracted using RIPA lysis buffer without SDS (150 mM NaCl, 5 mM EDTA, 50 mM Tris, pH 8.0, 0.5% sodium deoxycholate, and 1% NP-40) supplemented with 1% protease inhibitor cocktail (C0001; TargetMol). IP was performed using Anti-FLAG M2 Magnetic Beads (M8823; Sigma-Aldrich) as per the manufacturer’s protocol. The immunoprecipitated samples were eluted with 2× SDS sample buffer and boiled for 5 min at 95°C. The samples were then subjected to denaturation, SDS-PAGE, and immunoblotting analysis.

### PLA

5 × 10^4^ CHL-1 and A375 cells were cultured in Nunc Lab-Tek Chamber Slide (177402; Thermo Fisher Scientific). The cells were treated with DMSO, 500 nM celastrol, and 500 nM NR-V04 for 16 h with MG132 inhibitor. The PLA was conducted with Duolink In Situ Red Starter Kit Mouse/Rabbit (DUO92101; Sigma-Aldrich) following the standard protocol. Additionally, a non-stain cell group was included as a negative control to account for background signal during PLA assay. The signal was detected using a confocal microscope (LSM 710; Zeiss). Primary antibody: NR4A1 (HPA059742, 2 μg/ml, isotype-rabbit; Sigma-Aldrich) and VHL (MA5-13940, 2 μg/ml, isotype-mouse; Thermo Fisher Scientific).

### Quantitative PCR (qPCR)

Total RNA was extracted from cells using RNeasy Kits (74004; QIAGEN) following the manufacturer’s instructions. cDNA synthesis was performed using SuperScript III Reverse Transcriptase (18080044; Thermo Fisher Scientific) with 100 ng RNA as the template. Amplification of cDNA was carried out using 2× PowerUP SYBR Green Master Mix (A25778; Thermo Fisher Scientific). Each reaction was conducted in triplicates and results were normalized to the expression of a housekeeping gene. Primer (Eurofins): human *β-actin* (forward sequence: 5′-CAC​CAT​TGG​CAA​TGA​GCG​GTT​C-3′; reverse sequence: 5′-AGG​TCT​TTG​CGG​ATG​TCC​ACG​T-3′) and human *NR4A1* (forward sequence: 5′-GGA​CAA​CGC​TTC​ATG​CCA​GCA​T-3′; reverse sequence: 5′-CCT​TGT​TAG​CCA​GGC​AGA​TGT​AC-3′).

### PK study of NR-V04

The PK study of NR-V04 was done by Bioduro Inc. on three healthy male C57BL/6 mice weighing between 20 and 25 g in each group. Test compound (i.v.) was dissolved in 5% DMSO/3% tween 80 in PBS with 0.5 mEq 1N HCl and was administered (*n* = 3 per group) at a dose level of 2 mg/kg (concentration: 0.4 mg/ml). Test compounds (i.p.) were dissolved in 5% DMSO/3% tween 80 in PBS with 2 mEq 1N HCl (concentration 4 mg/kg) and were administrated at a dose level of 20 mg/kg. Plasma samples (i.v.) were collected from the orbital plexus at 0.083, 0.25, 0.5, 1.0, 2.0, 4.0, 8.0, and 24 h, and plasma samples (i.p.) were collected from the orbital plexus at 0.25, 0.50, 1.0, 2.0, 4.0, 8.0, and 24 h after dosing. The drug concentrations in the samples were quantified by LC/tandem MS. PK parameters were calculated from the mean plasma concentration by non-compartmental analysis.

### Animal experiments

All animal studies were approved by the University of Florida Institutional Animal Care and Use Committee (IACUC) under protocol 202110399. All animals were housed in a pathogen-free Association for Assessment and Accreditation of Laboratory Animal Care–accredited facility at the University of Florida and performed in accordance with IACUC guidelines. The room temperature is 21.1–23.3°C with an acceptable daily fluctuation of 2°C. Typically the room is 22.2°C all the time. The humidity set point is 50% but can vary ± 15% daily depending on the weather. The photoperiod is 12:12 and the light intensity range is 15.6–25.8 foot-candle.

7–9-wk-old C57BL/6J, B6.129S2-*Ighm*^tm1Cgn^/J (002288), B6.129S2-*Nr4a1*^*tm1Jmi*^/J (*NR4A1*^−/−^, 006187), and NOD-SCID interleukin-2 receptor gamma null (NSG) mice were purchased from Jackson Laboratories. For all studies involving NR-V04 and celastrol, the compounds were formulated in 50% phosal PG, 45% miglyol 810N, and 5% polysorbate 80 and administered via i.p. injection.

For syngeneic tumor models, 5 × 10^5^ cells MC38, Yumm1.7, and Yummer1.7, 1 × 10^5^ cells of B16F10 were resuspended in PBS and implanted into 7–9-wk-old mice subcutaneously. For the tumor inhibition experiment, mice were treated with 1.8 mg/kg NR-V04 or 0.75 mg/kg celastrol by i.p. injection twice weekly. For the immune profile experiment, once tumors reached 0.5 cm in diameter, mice were treated with 1.8 mg/kg NR-V04 by i.p. injection twice weekly. For the toxicity experiment, male and female non-tumor-bearing mice were treated with 2 mg/kg NR-V04 for two doses in the first week and 5 mg/kg for another two doses in the second week. Blood samples were taken from the submandibular (facial) vein before each treatment and 42 days later for hematological analysis, and body weight was measured twice per week up to day 42. Mice were euthanized in accordance with the IACUC protocol once the largest tumors reached 2 cm in diameter, and tissues were collected for further analysis.

### B cell proliferation assay

B cells were isolated from the spleen by using EasySep Mouse B cell Isolation Kit (19854; STEMCELL) and cultured in RPMI supplemented with 0.1 M non-essential amino acids (11140050; Thermo Fisher Scientific), 1 mM sodium pyruvate (11360070; Thermo Fisher Scientific), 1 mM Glutamax, 10 mM HEPES (15630080; Thermo Fisher Scientific), 0.1% 2-mercaptoethanol (21985023; Thermo Fisher Scientific), 10% FBS (F2442; Sigma-Aldrich) and 100 U/ml penicillin, and 100 µg/ml streptomycin (P4333; Sigma-Aldrich). B cells were stained with 5 nM CellTrace Violet Cell (C34557; Thermo Fisher Scientific) for 8 min in a 37°C water bath. B cells were plated at a concentration of 5 × 10^5^ cells per 200 μl in round-bottom 96-well plates treated with 0.1% B16F10 melanoma cell lysis or 10 µg/ml goat anti-mouse IgM F(ab′)2 (115-006-020; Jackson ImmunoResearch). B cells were treated with NR-V04 or DMSO for 24 h and collected for flow cytometry analysis.

### T cell activation and exhaustion in vitro

We conducted experiments involving the isolations and treatments of CD8^+^ T cells from both OT-1 mice (C57BL/6-Tg(TcraTcrb)1100Mjb/J; Jackson Laboratories) and T cells from human PBMCs. CD8^+^ T cells were isolated from OT-1 mice using the EasySep Mouse CD8 T Cell Isolation Kit, followed by daily treatment with 10 ng/ml ovalbumin (257–264) (S7951-1MG; Sigma-Aldrich) for specified durations. After 3 days of ovalbumin treatment, OT-1 CD8^+^ T cells were activated, and after 7 days of treatment, they reached a state of exhaustion. Human T cells were isolated from PBMCs using the EasySep Human CD3 Positive Selection Kit II (17851; STEMCELL) and cultured with Dynabeads (Cell/Dynabeads: 1:1, A56992; Thermo Fisher Scientific) for 3 days to induce activation and for 7 days to induce exhaustion. Both mouse and human T cells received a treatment of 500 nM NR-V04 2 days prior to harvesting. To characterize the T cell subtypes, we employed specific flow cytometry antibodies and conducted flow cytometry analysis.

Human blood and tissues were also collected from patients undergoing surgical resection after informed consent and were supplied as deidentified samples to Dr. Zhang’s laboratory, approved by the University of Florida Institutional Review Board (IRB201903411).

### CD8^+^ T cell depletion assay

We administered 100 μg of anti-mouse CD8α antibody (Clone: 53-6.7; Bio X Cell) or rat IgG2a isotype control to each mouse every 4 days via i.p. injection. This treatment regimen was initiated on day 7 after the inoculation of Yummer1.7 cancer cells. Toward the end of the experiment, we collected blood samples from mice in both treatment and control groups and assessed the efficiency of CD8^+^ T cell depletion using flow cytometry analysis.

### Flow cytometry

Mouse tumors were excised and ∼200 mg of tumor tissue was enzymatically and mechanically digested using the mouse Tumor Dissociation Kit (Miltenyi Biotec) to obtain a single-cell suspension. Human tumor samples and sections were enzymatically and mechanically digested using the human Tumor Dissociation Kit (Miltenyi Biotec) to obtain single-cell suspension. Red blood cells were lysed using ACK lysis buffer and mononuclear cells were isolated by density gradient using SepMate Tubes (StemCell Technologies) and Lymphoprep density gradient media (StemCell Technologies). Mouse cells were then washed and incubated with combinations of the following antibodies: anti-mouse CD62L-BV785 (clone MEL-14), anti-mouse MHCII I-A/I-E-BB515 (Clone 2G9, 1:400; BD Biosciences), anti-mouse CD11B-PEdazzle 594 (clone M1/70, 1:200), anti-mouse CD45-AF532 (clone 30F.11), anti-mouse CD3-APC/Cy7 (clone 17A2), anti-mouse CD8-BV510 (clone 53-6.7), anti-mouse CD4-BV605 (clone GK1.5), anti-mouse NK1.1-AF700 (clone PK136), anti-mouse/human CD45R/B220-BV 570 (clone RA3-6B2), anti-mouse CD138-BV711 (clone 281-2), anti-mouse IgD-BV711 (clone 281-2), anti-mouse IgD-PE-Cy7 (clone 11-26c.2a), anti-mouse Ly6G-FITC (clone IA8), anti-mouse Ly6C-BV711 (clone HK1.4), anti-mouse CD38-PerCP/Cy 5.5 (clone 90) anti-mouse IgM-AF488 (clone RMM-1), anti-mouse CD25-PE-Cy5 (clone PC61) plus zombie red (cell viability; Thermo Fisher Scientific), and mouse FcR blocker (anti-mouse CD16/CD32, clone 2.4G2; BD Biosciences). After surface staining, cells were fixed and permeabilized using the FOXP3/Transcription Factor Staining Buffer Set (eBioscience). Cells were stained with a combination of the following antibodies: anti-mouse FOXP3-efluor 450 (clone FJK-16S, 1:50; eBioscience), anti-mouse Granzyme B-BV421 (clone GB11), and anti-mouse NR4A1-PE (clone 12.14; eBioscience). Human cells were stained with a combination of the following antibodies: anti-human CD45-BV510 (clone H130), anti-human CD3-AF700 (clone HIT5a), anti-human CD4-BV421 (clone OKT4), anti-human CD8-BV711 (clone RPA-T8), anti-human CD127-BV605 (clone A019DS), anti-human CD25-PE-Cy7 (clone MA251) plus FVD-eFluor-780 (eBioscience), and human FcR blocking reagent (StemCell Technologies). Cells were washed then fixed and permeabilized using the eBioscience FOXP3/Transcription Factor Staining Buffer Set. Cells were further stained with a combination of the following antibodies: anti-human FOXP3-FITC (clone 206D) and anti-human NR4A1-PE (clone D63C5; Cell Signaling). Flow cytometry was performed on a three laser Cytek Aurora Cytometer (Cytek Biosciences) and analyzed using FlowJo software (BD Biosciences). All antibodies are from BioLegend, unless otherwise specified. Most antibodies were used at 1:100 dilution for flow cytometry, unless otherwise specified.

### Hematology analysis

Mouse blood was prepared in microcentrifuge tubes containing PBS with 10 mM EDTA. Blood indices were analyzed using an automated hematology analyzer (Element HT5; Heska).

### H&E staining

For H&E staining, mouse tissues were fixed in 10% formalin (SF98-4; Thermo Fisher Scientific) processed in the University of Florida Molecular Pathology Core. H&E staining was conducted using standard procedures. The sections were hydrated through xylene and a series of ethanol, followed by staining with H&E on slides.

### Statistics

Graphs and statistical analyses were performed using Prism software (GraphPad Software) unless otherwise specified. Tumor growth curves were compared using a two-way analysis of variance (ANOVA). For comparisons involving three or more groups, a one-way ANOVA was conducted, followed by Dunnett’s multiple comparison test for specific group comparisons. Unpaired *t* tests were used to compare means between two groups.

### Online supplemental material

We include five supplementary figures. Fig. S1 highlights an inverse correlation between *NR4A1* expression and T cell activation effector molecules (*IFNG*, *GZMB*, and *PRF1*) in melanoma, with low *NR4A1* expression associated with enriched immune activation pathways. Fig. S2 shows that NR-V04 induces NR4A1 degradation in human and mouse melanoma cell lines without affecting NR4A2 and NR4A3, and forms a ternary complex in CHL-1 cells. Fig. S3 focuses on the effect of celastrol and NR-V04 treatments in different mouse tumor models, including flow cytometry gating strategies for immune cell analysis. Fig. S4 reveals NR-V04’s role in regulating B and T cell populations in melanoma, demonstrating significant proliferation and activation changes. Fig. S5 confirms the minimal toxicity of NR-V04, showing no significant changes in various hematologic profiles in treated mice.

## Supplementary Material

SourceData F3 is the source file for Fig. 3.

SourceData F4 is the source file for Fig. 4.

SourceData F5 is the source file for Fig. 5.

SourceData F6 is the source file for Fig. 6.

SourceData FS2 is the source file for Fig. S2.

## Data Availability

We utilized multiple GEO datasets including GSE120575, GSE148190, GSE158803, and GSE12163. The human TCGA melanoma data cohort was downloaded from the TCGA portal for analysis. The code used in our analysis is available on GitHub (https://github.com/Levy0803/NR4A1).
