# Supplementary figures and images for: PROTAC-mediated NR4A1 degradation as a novel strategy for cancer immunotherapy
Source: J Exp Med. 2024 Feb 9;221(3):e20231519. doi: 10.1084/jem.20231519 (PMC10857906; doi:10.1084/jem.20231519)

C

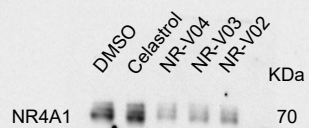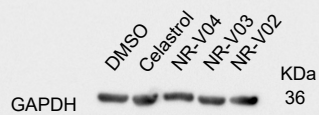

Supplement: SourceData F3 — is the source file for Fig. 3. [file JEM_20231519_SourceDataF3.pdf]

Figure 4A

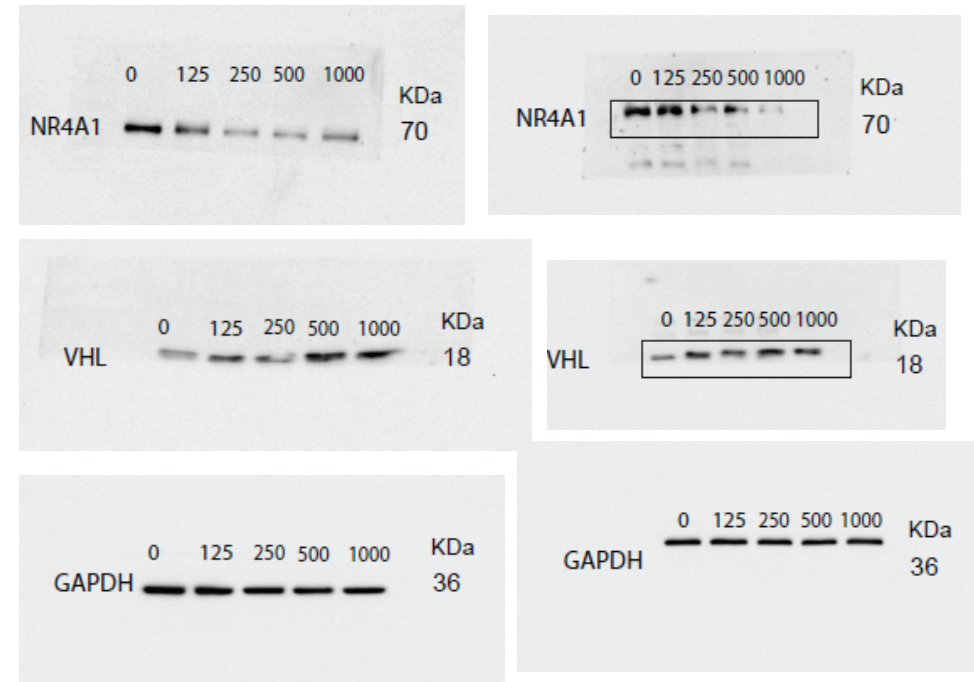

Figure 4B

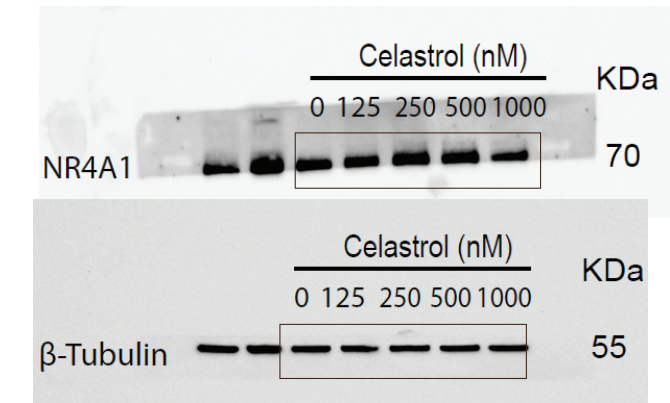

Figure 4C

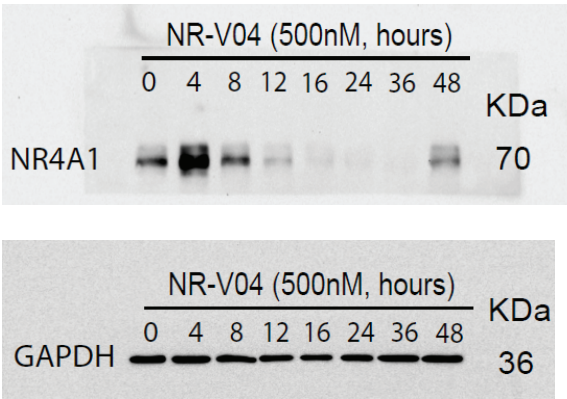

Figure 4D

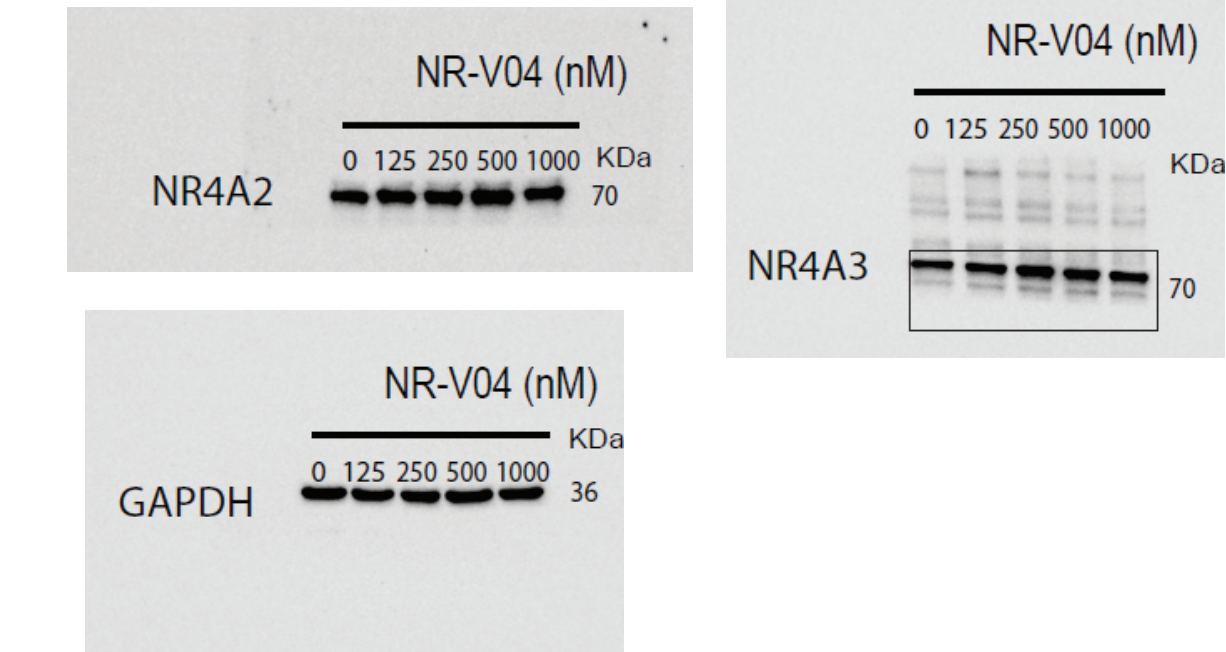

Supplement: SourceData F4 — is the source file for Fig. 4. [file JEM_20231519_SourceDataF4.pdf]

Figure 5B

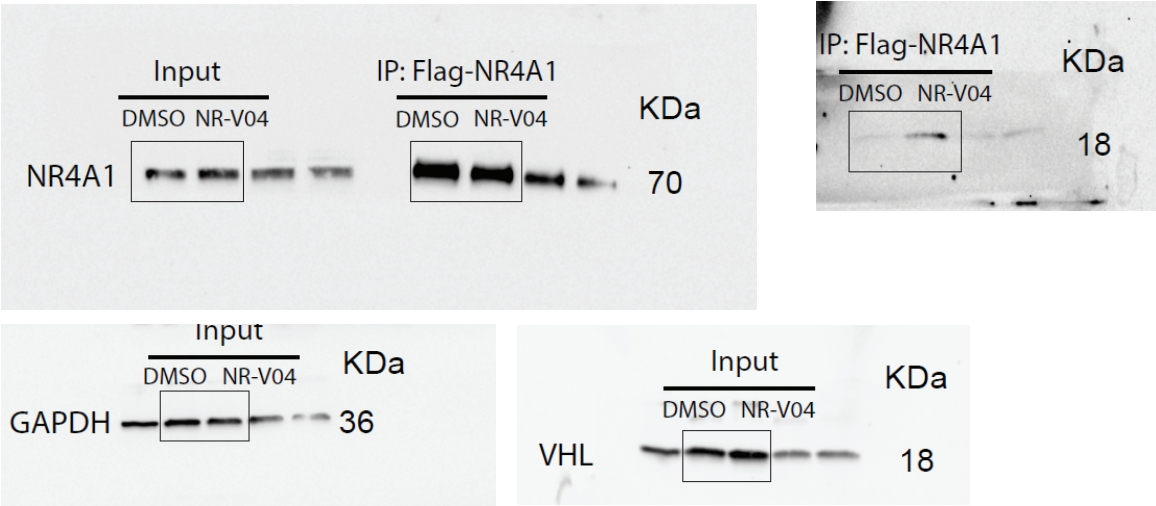

Figure 5C

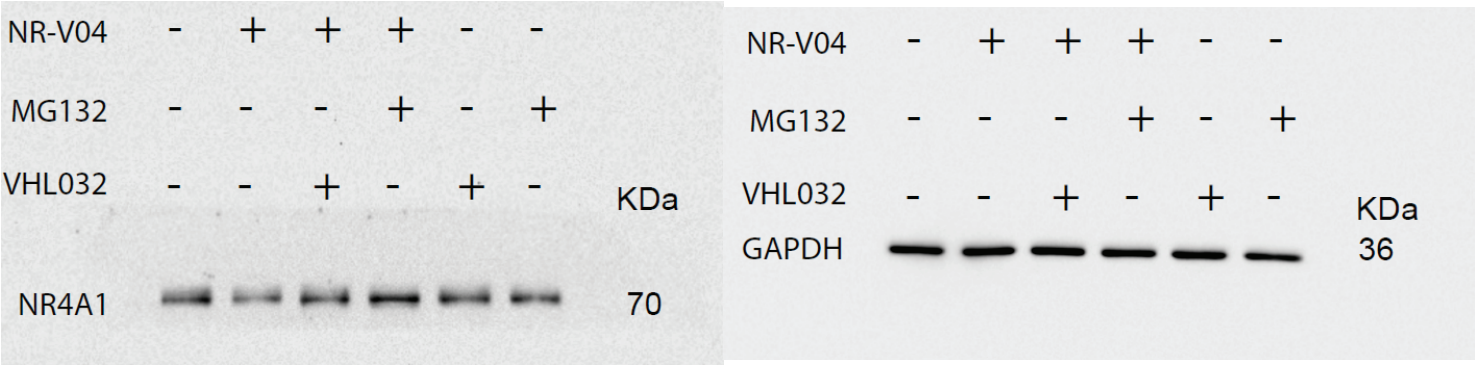

Supplement: SourceData F5 — is the source file for Fig. 5. [file JEM_20231519_SourceDataF5.pdf]

Figure S2A

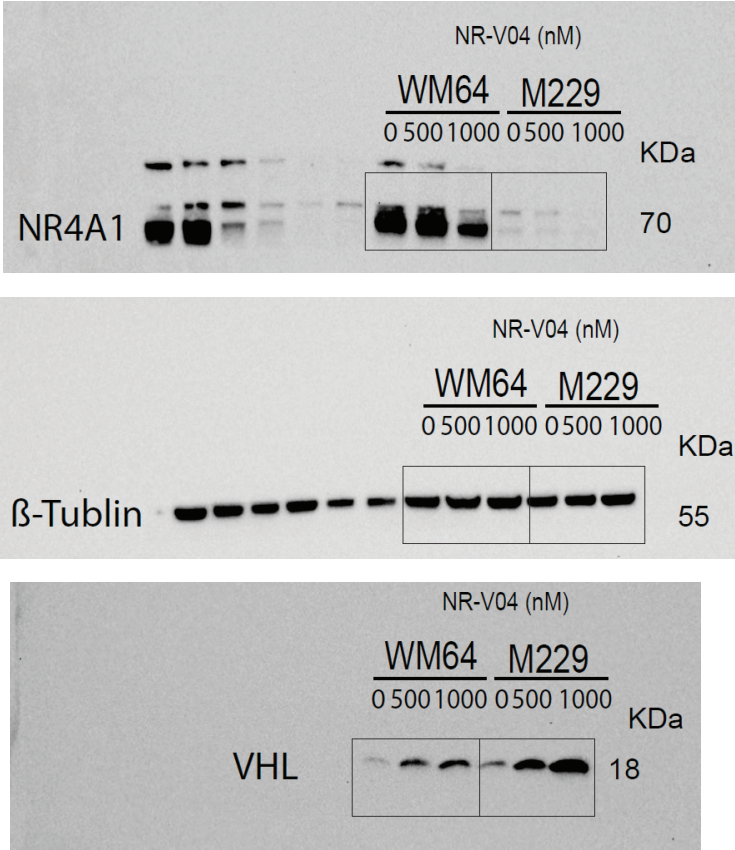

Figure S2B

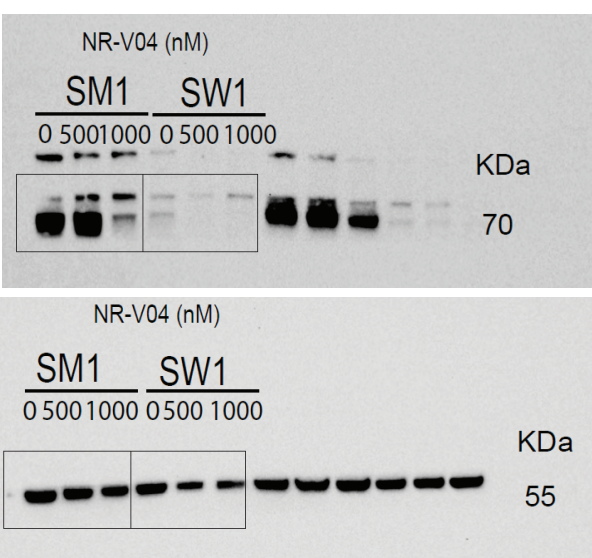

Figure S2C

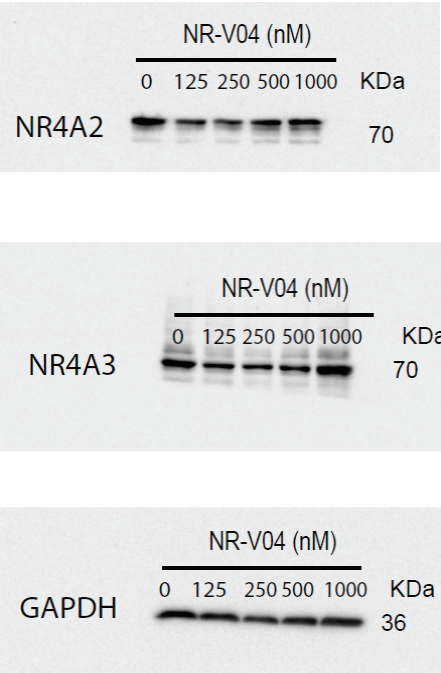

Figure S2F

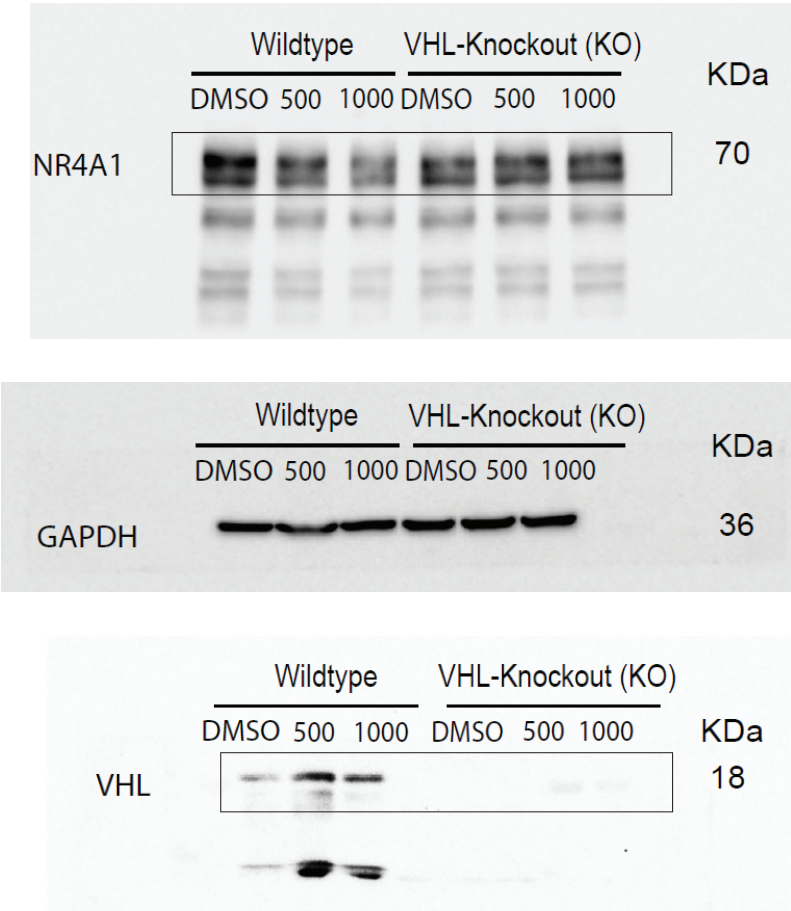

Supplement: SourceData FS2 — is the source file for Fig. S2. [file JEM_20231519_SourceDataFS2.pdf]
