# Supplementary material for: PROTAC-mediated NR4A1 degradation as a novel strategy for cancer immunotherapy
Source: J Exp Med. 2024 Feb 9;221(3):e20231519. doi: 10.1084/jem.20231519 (PMC10857906; doi:10.1084/jem.20231519)

Figure 6B

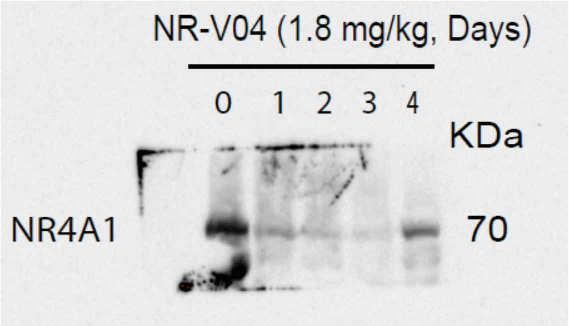

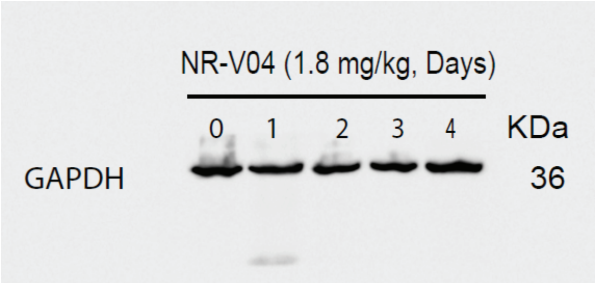

Figure 6C

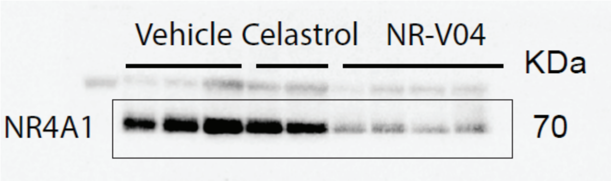

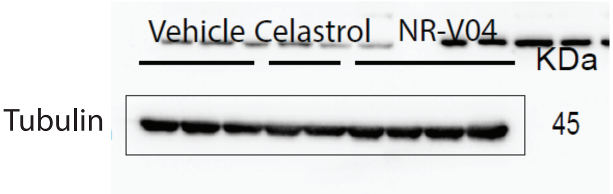

Supplement: SourceData F6 — is the source file for Fig. 6. [file JEM_20231519_SourceDataF6.pdf]
